# Dietary potassium stimulates *Ppp1Ca-Ppp1r1a* dephosphorylation of kidney NaCl cotransporter and reduces blood pressure

**DOI:** 10.1172/JCI158498

**Published:** 2023-11-01

**Authors:** P. Richard Grimm, Anamaria Tatomir, Lena L. Rosenbaek, Bo Young Kim, Dimin Li, Eric J. Delpire, Robert A. Fenton, Paul A. Welling

**Affiliations:** 1 Department of Medicine (Nephrology), Johns Hopkins University School of Medicine Baltimore, Maryland, USA.; 2 The LeDucq Potassium in Hypertension Research Network of Excellence is detailed in Supplemental Acknowledgments.; 3 Department of Biomedicine, University of Aarhus, Aarhus, Denmark.; 4 Department of Anesthesiology, Vanderbilt University Medical Center, Nashville, Tennssee, USA.; 5 Department of Physiology, Johns Hopkins University School of Medicine Baltimore, Maryland, USA.

**Keywords:** Nephrology, Hypertension, Phosphoprotein phosphatases, Transport

## Abstract

Consumption of low dietary potassium, common with ultraprocessed foods, activates the thiazide-sensitive sodium chloride cotransporter (NCC) via the with no (K) lysine kinase/STE20/SPS1-related proline-alanine–rich protein kinase (WNK/SPAK) pathway to induce salt retention and elevate blood pressure (BP). However, it remains unclear how high-potassium “DASH-like” diets (dietary approaches to stop hypertension) inactivate the cotransporter and whether this decreases BP. A transcriptomics screen identified *Ppp1Ca*, encoding PP1A, as a potassium-upregulated gene, and its negative regulator *Ppp1r1a*, as a potassium-suppressed gene in the kidney. PP1A directly binds to and dephosphorylates NCC when extracellular potassium is elevated. Using mice genetically engineered to constitutively activate the NCC-regulatory kinase SPAK and thereby eliminate the effects of the WNK/SPAK kinase cascade, we confirmed that PP1A dephosphorylated NCC directly in a potassium-regulated manner. Prior adaptation to a high-potassium diet was required to maximally dephosphorylate NCC and lower BP in constitutively active SPAK mice, and this was associated with potassium-dependent suppression of *Ppp1r1a* and dephosphorylation of its cognate protein, inhibitory subunit 1 (I1). In conclusion, potassium-dependent activation of PP1A and inhibition of I1 drove NCC dephosphorylation, providing a mechanism to explain how high dietary K^+^ lowers BP. Shifting signaling of PP1A in favor of activation of WNK/SPAK may provide an improved therapeutic approach for treating salt-sensitive hypertension.

## Graphical abstract

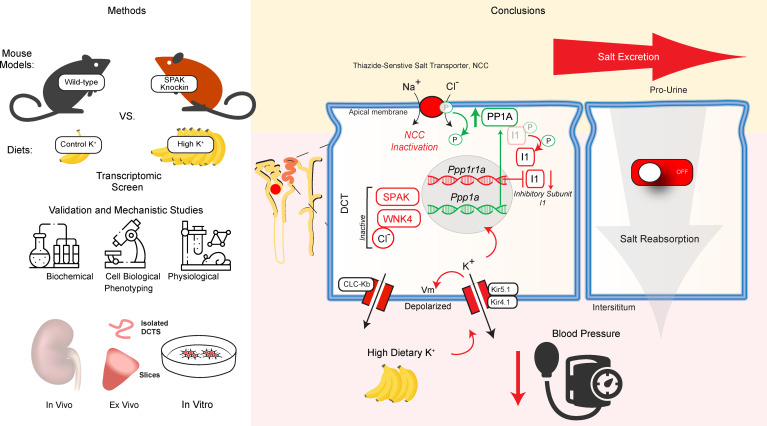

## Introduction

According to the WHO, hypertension is the single greatest contributor to premature death and disability in the world (1). High salt (NaCl) consumption has long been a major culprit, but growing evidence indicates that dietary potassium has an equally important role. Low dietary potassium increases salt sensitivity and blood pressure (BP) (2–4), whereas high potassium decreases salt sensitivity and lowers BP (5, 6). A recent large-scale clinical trial revealed that substituting 25% of dietary sodium intake with potassium lowered BP and the risk of adverse cardiovascular events and death (7). Although the mechanisms underlying the effects of potassium are undoubtedly multifactorial and complex, recent studies have highlighted the potential involvement in the kidney of a salt-regulatory pathway called the “potassium switch” (8–10).

The existence of the switch pathway was first suggested when activating mutations in the with no (K) lysine kinases (WNKs) were identified as a cause of familial hyperkalemic hypertension (FFHt) (11), which is also known as Gordon syndrome, or pseudohypoaldosteronism type II. Subsequent mechanistic studies revealed that WNKs in the distal convoluted tubule (DCT) are part of a kinase cascade that is responsible for adjusting the activity of the thiazide-sensitive sodium chloride cotransporter (NCC, encoded by the *SLC12A3* gene) to maintain sodium and potassium balance in the face of widely varied potassium intake (8, 9). The “switch” metaphor comes from the ability of potassium to turn the signaling pathway “on” or “off” over the narrow physiologic range of extracellular potassium concentrations, depending on physiological need.

In recent years, considerable progress has been made in understanding how dietary potassium deficiency activates the NCC. According to current understanding, the activating arm of the switch pathway consists nominally of a basolateral membrane potassium channel complex (Kir4.1/Kir5.1 encoded by the *KCNJ10/16* genes) (12–15), WNK4 (16, 17), and the STE20/SPS1-related proline-alanine–rich protein kinase (SPAK) (18–21). When activated by WNK4, SPAK binds to and phosphorylates NCC, thereby activating transport. The inactive conformation of WNKs is stabilized by chloride (22, 23) and potassium (24). Upon physiological reductions in extracellular potassium levels, Kir4.1/Kir5.1-dependent membrane hyperpolarization drives chloride from the cell (22, 25, 26), which lowers intracellular chloride enough to relieve the WNK4 inhibition (27) and activate the kinase cascade to phosphorylate NCC (26, 28, 29).

In contrast to our understanding of switch activation, the mechanism by which increased dietary potassium turns the switch off (30–33) is unclear. Increasing plasma potassium by intravenous potassium infusion is sufficient to rapidly reduce NCC phosphorylation (34). It has been generally assumed that basal protein phosphatase activity dephosphorylates the WNK and SPAK cascade once Cl^–^ binds to and stabilizes the inactive conformation of WNK, terminating kinase signaling. However, this cannot explain how NCC is rapidly dephosphorylated (33, 34). Unlike kinase activation, potassium-induced NCC dephosphorylation is not dependent on changes in intracellular Cl^–^ (27, 32). Several phosphatases have been implicated in limiting phosphorylation through the WNK-SPAK-NCC cascade (35–39), but it is unknown if they are physiologically activated in the DCT by potassium. Furthermore, it is unclear which step(s) potential phosphatases function in the potassium switch cascade and whether they directly dephosphorylate NCC. Because calcineurin (40) inhibitors can increase NCC phosphorylation (39), calcineurin has been considered a likely candidate. However, recent studies suggest that calcineurin may chiefly control WNK4 rather than regulate NCC phosphorylation directly (40), and loss of calcineurin signaling using DCT-specific KO of the regulatory B subunit does not block K^+^-dependent dephosphorylation of NCC (41). By contrast, protein phosphatase 1 may control NCC phosphorylation directly (31). Picard et al. reported that protein phosphatase inhibitory subunit 1 (I1) is enriched in the DCT, and KO studies revealed that it is necessary to maintain NCC phosphorylation and prevent hypotension (37), whereas studies by Penton et al. showed that PKA-dependent phosphorylation of I1 and subsequent inhibition of PP1 causes an increase in NCC phosphorylation (31), which is especially relevant for adrenergic activation of salt retention and salt-sensitive hypertension (42). In the present study, we sought to determine how potassium dephosphorylates NCC and explore whether this pathway decreases BP.

## Results

**Figure 1:**
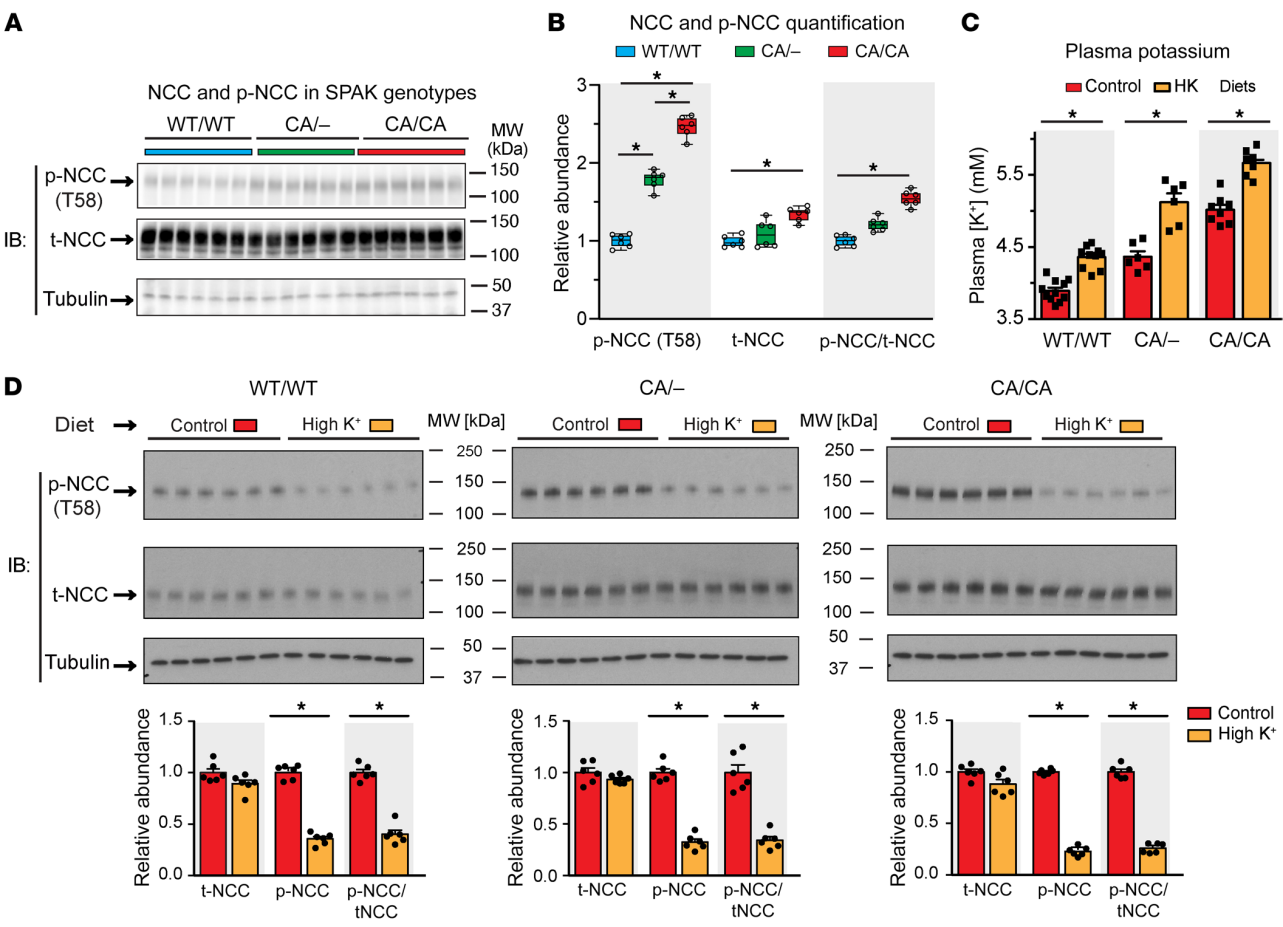
Consumption of potassium-rich diet triggers direct dephosphorylation of NCC. (**A**) Immunoblot (IB) and (**B**) quantitative summary of p-NCC (T58) and t-NCC in control mice (blue) homozygous for WT SPAK (WT/WT, blue), heterozygous CA-SPAK mice (CA/–, green), and homozygous CA-SPAK mice (CA/CA mice, red). Each lane/data point represents a separate mouse. (**B**) Immunoblot and quantitative summary of p-NCC (T58) and t-NCC levels in mice randomized to the control diet (orange bars) or the high-potassium diet for 4 days (red bars). Each lane/data point represents a separate mouse. (**C**) Plasma potassium (K^+^) levels for mice of the 3 different SPAK genotypes randomized to the control diet (orange bars) or the high-potassium diet for 4 days (red bars). *P < 0.05. Statistical significance across genotypes was assessed by 1-way ANOVA followed by Tukey’s post hoc test. In **C** and **D**, potassium responses within a genotype were assessed by paired *t* test. *n* = 6. Values are the mean ± SEM.

To determine whether a potassium-regulated protein phosphatase pathway modulates NCC, we used a genetic strategy to isolate the direct effects of NCC dephosphorylation from the inactivation of the upstream kinase cascade. This was accomplished by inserting activating mutations in SPAK and specifically inducing expression of the constitutively active kinase only in the DCT. As reported previously (43), homozygous DCT-specific constitutively active (CA) SPAK mice (CA/CA) exhibit robust NCC phosphorylation at the key activation site, threonine 58 (T58) (Figure 1, A and B), despite hyperkalemia (Figure 1C). Mice harboring a CA-SPAK allele and a null SPAK allele (CA/–) are also able to sustain high levels of T58 phosphorylation (Figure 1, A and B) despite higher plasma potassium levels than those detected in the control mice (Figure 1C). Following ingestion of a potassium-rich diet for 4 days, the homozygous and heterozygous CA-SPAK mice displayed a comparable decrease in phosphorylated NCC (p-NCC) at T58 comparable to that seen in control mice (Figure 1D), albeit at higher plasma potassium levels (Figure 1C). The ability of potassium to override constitutive SPAK phosphorylation of NCC is consistent with a protein phosphatase that directly dephosphorylates NCC.

**Figure 2:**
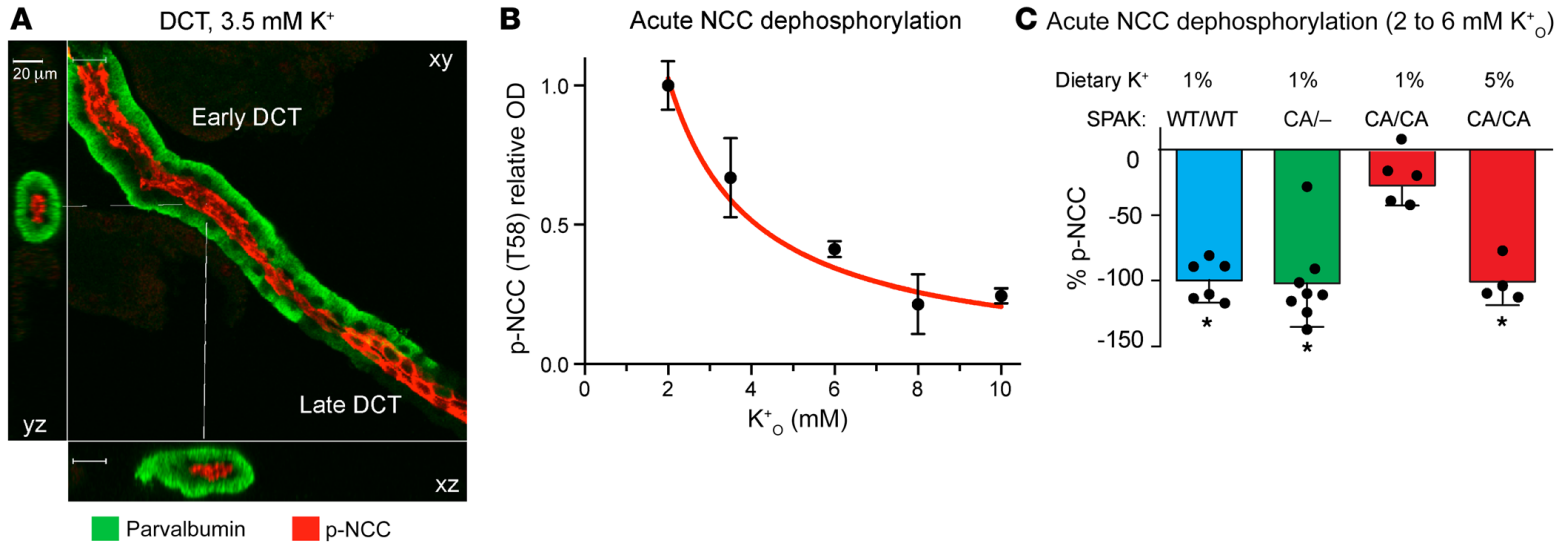
Potassium-dependent NCC dephosphorylation in isolated DCT overrides constitutively active SPAK. (**A**) Typical confocal image of an isolated DCT labeled with p-NCC (T58) (red) and anti-parvalbumin antibodies (green). Scale bar: 20 μm. (**B**) Abundance of p-NCC (T58) in WT mice over a titration of extracellular potassium concentrations (K^+^_o_), 20 minutes after potassium was elevated from a preconditioning medium containing 2.0 mM K^+^_o_. Shown are p-NCC pixel intensities at each K^+^_o_ relative to the maximum phosphorylation at 2.0 mM potassium. *n* >6 tubules per condition. (**C**) Acute NCC (T58) dephosphorylation, 20 minutes after K^+^_o_ was increased from 2 mM to 6 mM in control mice (WT/WT SPAK), heterozygous CA-SPAK mice (CA/–), and homozygous CA-SPAK mice (CA/CA) fed a control potassium (1%) or high-potassium (5%) diet for 4 days. Each dot represents a separate mouse. *P < 0.05, by 1-way ANOVA with Tukey’s post hoc test. Data are the mean ± SEM. *n* > 4 per group.

Dietary potassium loading has been proposed to stimulate the rapid dephosphorylation of NCC through a neurohormonal pathway that emanates from the gastrointestinal (GI) tract (33), a direct potassium-sensing mechanism in the DCT (32, 34), or both. To explore whether acute changes in extracellular potassium are sufficient to stimulate NCC dephosphorylation directly, we isolated DCTs from mouse kidneys and exposed the tubules to varying extracellular potassium levels. We identified early DCTs (DCT1) by parvalbumin labeling and evaluated p-NCC (T58) levels by semiquantitative microscopy following antibody labeling (Figure 2A). In tubules isolated from control mice, an increase in extracellular potassium decreased p-NCC within 20 minutes in a dose-dependent manner (Figure 2B). The dephosphorylation response following an increase in extracellular potassium from 2 to 6 mM at 20 minutes was identical in control mice and heterozygous CA-SPAK mice fed a control diet, consistent with direct potassium-dependent dephosphorylation of NCC (Figure 2C). By contrast, NCC phosphorylation was unaffected by the acute increase in extracellular potassium in tubules from homozygous CA-SPAK mice fed a control diet (Figure 2C). However, the acute NCC dephosphorylation response was restored after the homozygous CA-SPAK mice were fed a high-potassium diet for 4 days (Figure 2C). These data indicate that increased dietary potassium consumption promotes protein phosphatase activity to overcome the dominant stimulatory effects of the CA-SPAK kinase on NCC phosphorylation in the early DCT. Similar regulation may occur in the much shorter late DCT (DCT2) segment, but we could not confirm this, as the segment proved too difficult to isolate reproducibly.

**Figure 3:**
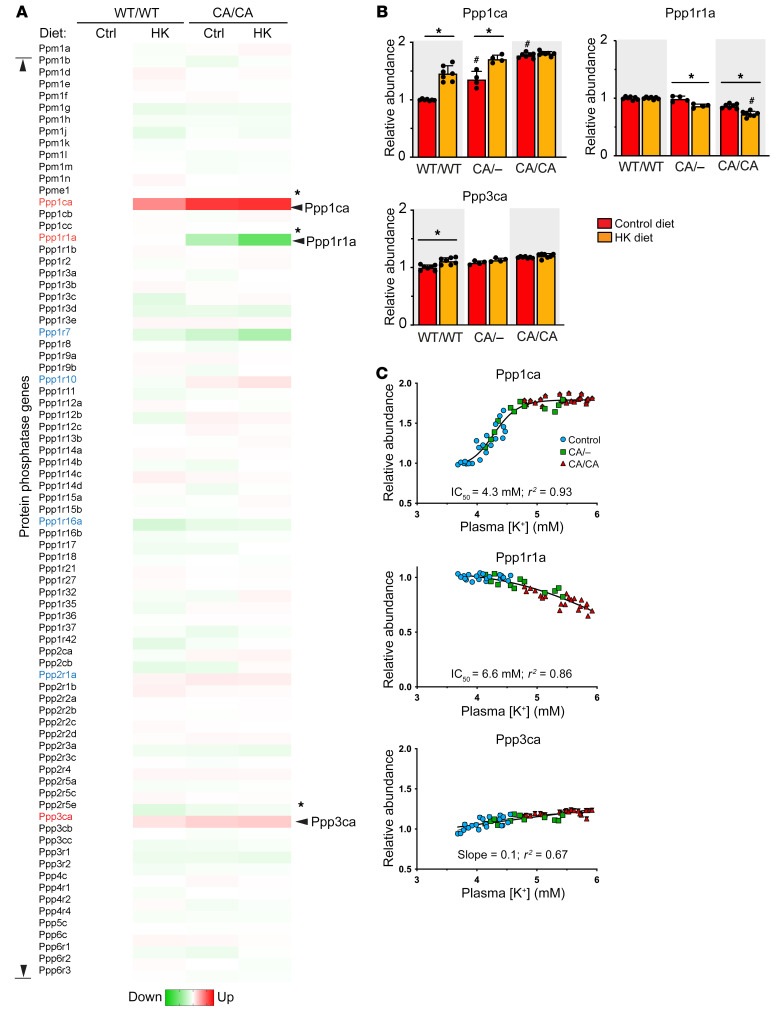
Dietary potassium differentially regulates *Ppp1Ca* and *Ppp1r1a* genes in the kidney. (**A**) Targeted protein phosphatase screen comparing all 71 serine/threonine phosphatases and regulatory subunits in the kidney. Heatmap shows the transcript abundance of each protein phosphatase (labeled on the left) relative to control mice (homozygous for WT SPAK, WT/WT) on a control diet. Control (WT/WT) and homozygous CA-SPAK mice (CA/CA) on control (Ctrl) and high-potassium diets (HK) are compared. Green indicates downregulated (Down); red indicates upregulated (Up). *n* = 7 mice per group. *P < 0.05, by 1-way ANOVA for all groups followed by Tukey’s comparison (genes whose expression significantly changed by at least 20% from WT/WT levels in mice on the control diet are indicated with a single asterisk and were selected for validation). (**B**) qPCR validation of potassium-dependent regulation of *Ppp1Ca* and *Ppp1R1a*. Each dot is a separate mouse. Transcript abundances are shown relative to control mice on the control diet (WT/WT SPAK). Statistical significance was assessed as above. *P < 0.05, statistical differences for diet effect within a genotype; ^#^*P* < 0.05, statistical difference between CA/– and CA/CA mice on a control diet versus WT/WT mice on a control diet. Data are the mean ± SEM. *n* >7. (**C**) Relative transcript abundance versus plasma potassium concentration. Best-fit lines (as determined by *F* test analysis) are shown. Ppp1Ca and Ppp1r1a lines are fit to abundance = maximum abundance/1 + (K_1/2_/PK^+^)^n^. Ppp3ca is fit to linear.

**Figure 4:**
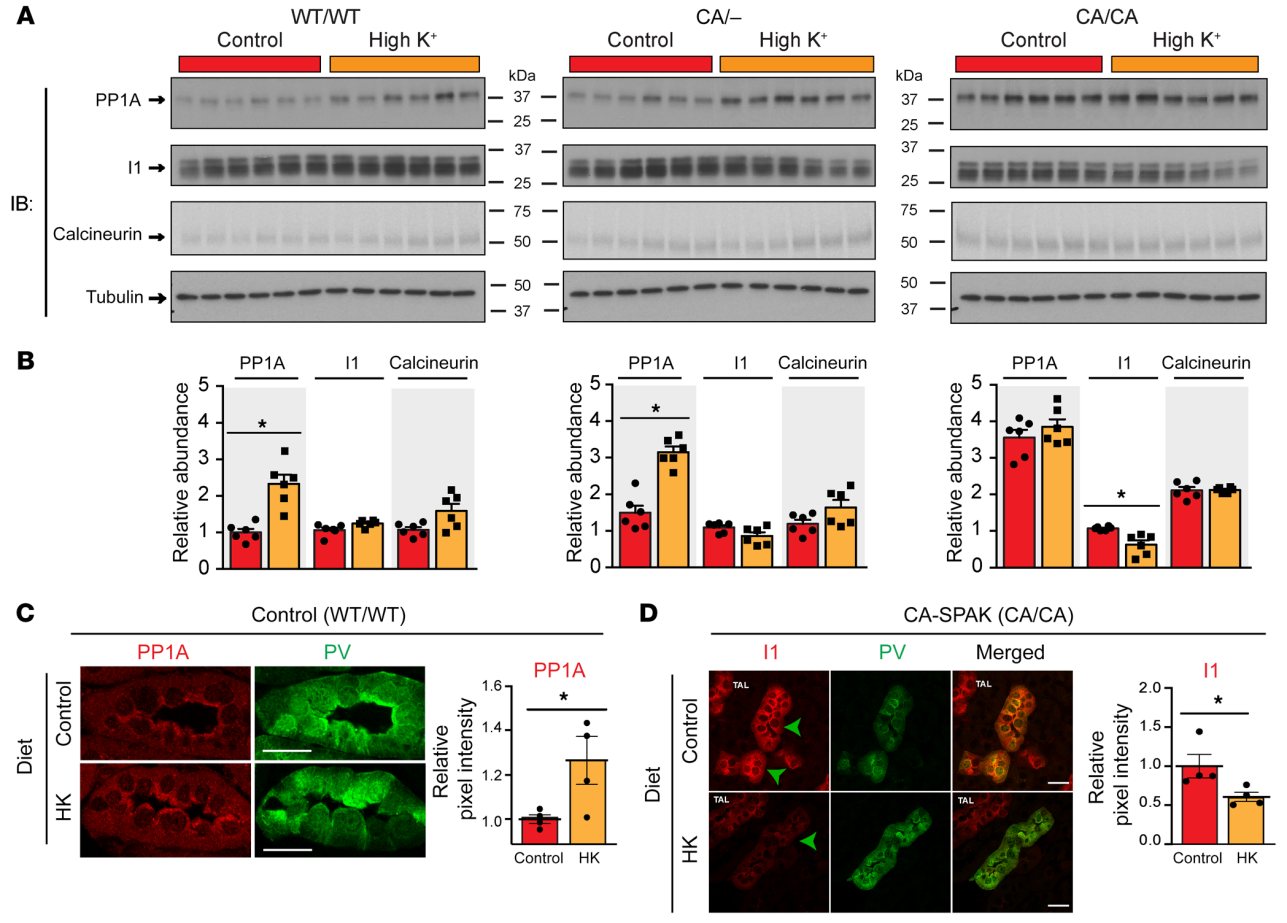
Consumption of a potassium-rich diet increases PP1 protein abundance and decreases I1 protein abundance in the DCT. (**A**) Immunoblot analysis of renal PP1, I1, and calcineurin in control mice (WT/WT SPAK), or mice with 1 (CA/–) or 2 (CA/CA) CA-SPAK alleles fed a control or high-potassium diet for 4 days. Each lane is from a separate mouse. (**B**) Quantitative summaries for **A**; shown is the average protein abundance of PP1, I1, and calcineurin for mice of each genotype on the control diet (orange) or the high-potassium diet (red). Data are relative to control mice on the control diet. (**C**) PP1A localization in control mice harboring 2 WT SPAK alleles (WT/WT). Representative images show PP1A (red) in DCT1, identified by parvalbumin (PV) labeling (green), from WT mice randomized to the control or high-potassium diet for 4 days. Scale bars: 15 μm. Graph shows quantitative image analysis of total cellular PP1A intensity (*n* = 4 mice/group, each data point represents the average intensity of >30 cells/mouse). *P < 0.05, by 2-tailed Student’s *t* test. (**D**) I1 localization in CA-SPAK mice harboring 2 CA-SPAK alleles (CA/CA). Representative images show I1 (red) in DCT1 from homozygous CA-SPAK (WT) mice randomized to the control or high-potassium diet for 4 days. Scale bars: 20 μm. Parvalbumin labeling (green) identified DCT1 (green arrowheads) from the cortical thick ascending limb (TAL), which also expressed I1. Graph shows quantitative image analysis of total cellular I1 intensity (4 mice/group, each data point represents the average intensity of >30 cells/mouse). Data are the mean ± SEM. *P < 0.05, by 2-tailed Student’s *t* test.

We performed a transcriptomic screen to identify protein phosphatase genes regulated by dietary potassium, profiling all 71 serine/threonine phosphatases and regulatory subunits known to be expressed in the kidney (18). We tested kidney cortex samples of WT and CA-SPAK mice on a control or high-potassium diet for 4 days. *Ppp1Ca* and *Ppp3Ca*, encoding catalytic protein phosphatase PP1A and calcineurin subunits, were identified as the most potassium-upregulated genes. *Ppp1r1a*, encoding a protein phosphatase–inhibitory subunit, was the only potassium-downregulated gene (Figure 3A and Supplemental Table 1; supplemental material available online with this article; https://doi.org/10.1172/JCI158498DS1). Subsequent validation analysis confirmed the changes in *Ppp1ca* and *Ppp1r1a* transcript levels (Figure 3, B and C) and their cognate proteins (Figure 4). Although *Ppp3ca* transcript expression significantly increased, the effect was only observed in WT mice (Figure 3B), was weakly dependent on plasma potassium [P_K+_] (Figure 3C), and did not translate to an increase in the cognate protein calcineurin (Figure 4).

The *Ppp1ca* transcript (Figure 3B) and its cognate protein PP1A (Figure 4, A and B) increased in control and heterozygous CA-SPAK mice on the high-potassium diet. We observed a tight positive correlation between *Ppp1ca* transcript abundance and P_K+_, as P_K+_ increased from approximately 3.8 to 4.8 mM (IC_50_ 4.3 mM) (Figure 3C). Immunofluorescence confocal microscopy revealed that the increase in PP1A occurred specifically in the DCT, and the change was most evident on or near the apical membrane (Figure 4C). In homozygous CA-SPAK mice, *Ppp1ca* (Figure 3, B and C) and PP1A (Figure 4, A and B) levels were elevated at their hyperkalemic baseline (~4.8 mM) compared with levels in normokalemic control mice and did not increase further after dietary potassium loading and an additional increase in P_K+_. Together, these data indicate the *Ppp1ca* gene and PP1A protein were tightly upregulated in the DCT over a physiological range of P_K+_ (Figure 3C), correlating with the titration of NCC dephosphorylation (see below).

**Figure 5:**
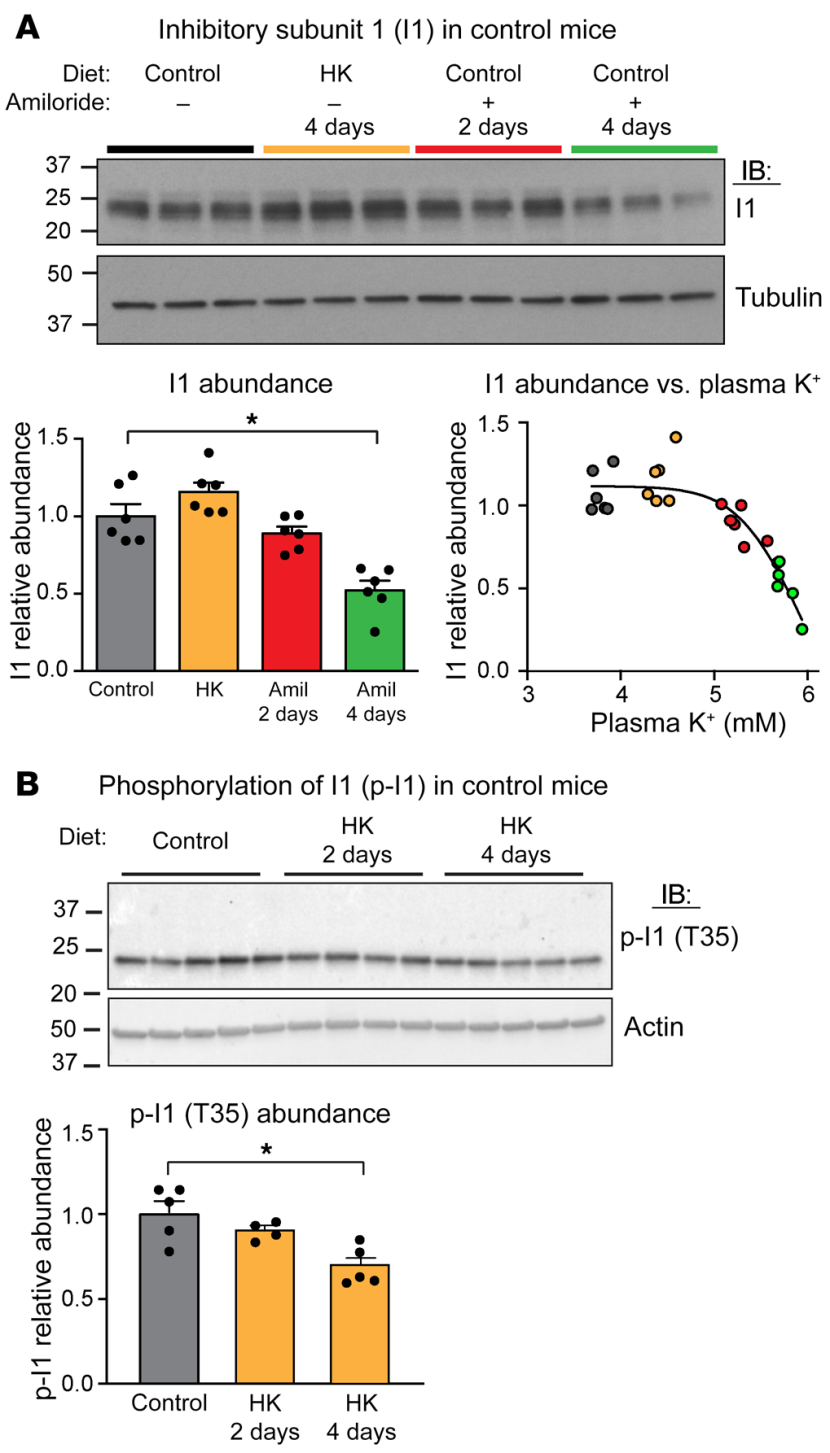
Potassium liberates PP1 from I1 by 2 mechanisms in control mice. (**A**) I1 abundance decreased as mice became hyperkalemic, as shown in the immunoblot of I1 in control mice on the control (gray) or high-potassium (orange) diet or in mice treated with amiloride (Amil) for 2 days (red) or 4 days (green). Quantitative summaries show the average I1 abundance and relative abundance versus plasma potassium. Each dot represents a separate mouse. (**B**) A high-potassium diet decreased I1 phosphorylation at T35 in WT mice. Immunoblot shows p-I1 (T35). Graph shows the quantitative summary of p-I1 relative abundance. *P < 0.05, by 1-way ANOVA followed by Dunnett’s multiple-comparison test (**A** and **B**). Data are the mean ± SEM. *n* = 6 (**A**); n = 4–5 (**B**).

With the high-potassium diet, *Ppp1r1* transcript levels (Figure 3B) decreased significantly in homozygous and heterozygous and CA-SPAK mice. Expression of the cognate protein I1 (44) decreased significantly in CA/CA mice but not in the WT/WT or heterozygous CA-SPAK mice (Figure 4, A and B). Confocal microscopy confirmed that I1 was reduced specifically in the DCTs of CA-SPAK mice on a high-potassium diet (Figure 4D). Because *Ppp1r1* transcripts decreased in the CA-SPAK mice when the P_K_ exceeded approximately 5 mM (Figure 3C), we tested whether potassium-dependent downregulation of *Ppp1r1* might have a hyperkalemic threshold in control mice by examining the effects of a high-potassium diet or amiloride, which reduces urinary potassium excretion and increases P_K+_. As shown in Figure 5A, the abundance of I1 decreased in control mice when P_K+_ rose above approximately 5 mM with amiloride treatment but not with modest changes in P_K+_ after increasing dietary potassium. By contrast, phosphorylation of I1 at T35 decreased with more modest changes in P_K+_ in control mice after 4 days on a high-potassium diet (Figure 5B). Because I1 binding to PP1A is dependent on T35 phosphorylation, I1 dephosphorylation is expected to increase PP1 phosphatase activity.

**Figure 6:**
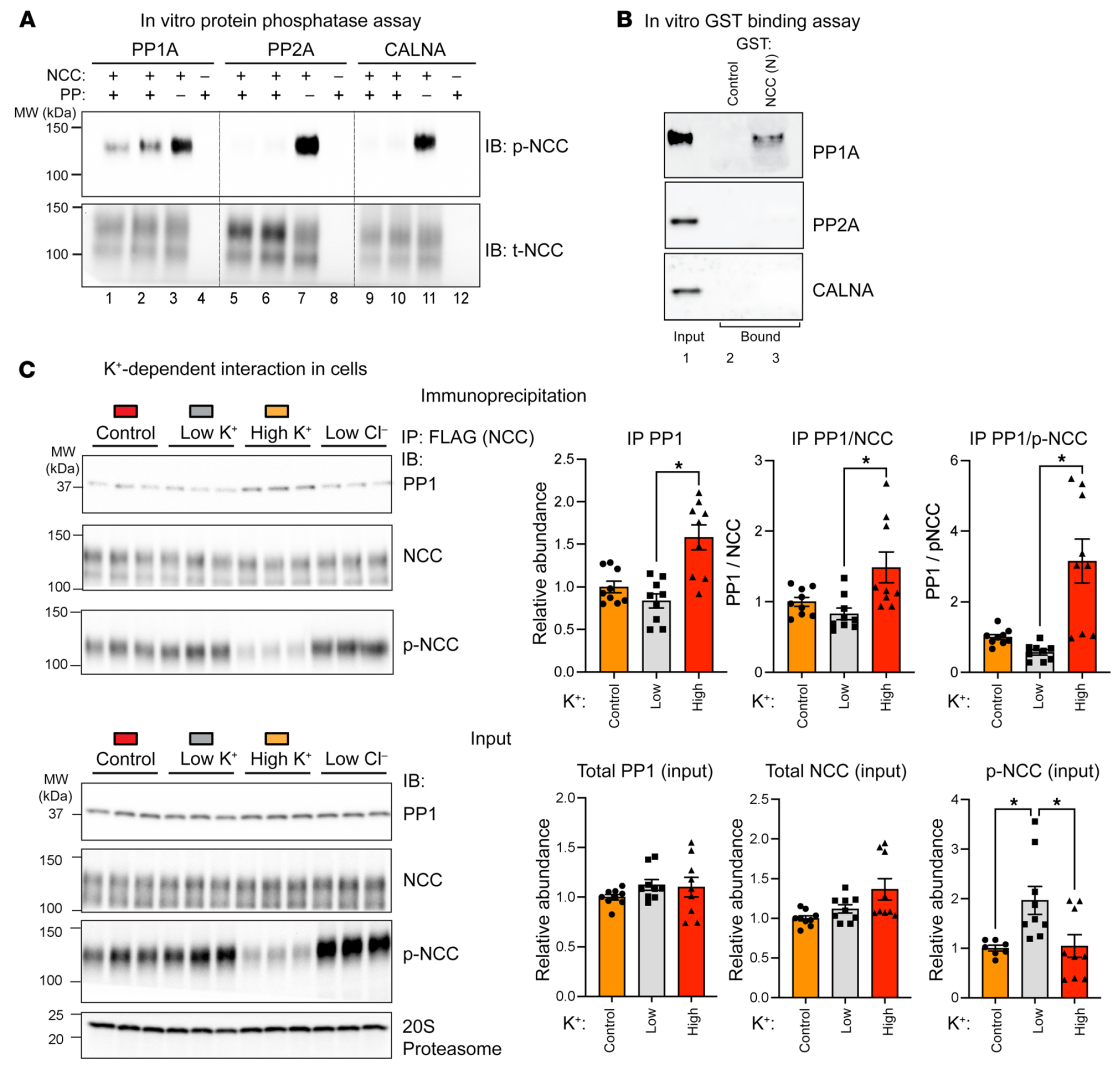
Potassium regulates PP1A interaction with and dephosphorylation of NCC. (**A**) In vitro protein phosphatase (PP) assay. Representative immunoblots of p-NCC and t-NCC are shown for assays of NCC with PP1A (lanes 1 and 2), PP2A (lanes 5 and 6), and calcineurin A (CALNA) or vehicle (lanes 3, 7, and 11). For these studies, NCC was isolated by IP with an anti-NCC antibody (lanes 1, 2, 3, 5, 6, and 7) and compared with the negative control IgG (lanes 4, 8, and 12). (**B**) PP1A preferentially interacted with NCC in glutathione-agarose affinity chromatography assays with the GST fusion protein of the NCC terminus GST-NCC-(N), but not the negative control GST alone. Shown are representative immunoblot binding assays with recombinant PP1A, PP2A, and CALNA. Input protein phosphatase (lane 1) is shown relative to GST-bound (lane 2) or GST-NCC-(N) (lane 3). (**C**) A greater amount of PP1A co-immunoprecipitated with NCC when the extracellular K^+^ concentration was high. Representative immunoblots show NCC, PP1A, and p-NCC (T58) in anti-FLAG immunoprecipitated samples relative to input from FLAG-tagged NCC-expressing MDCKI cells incubated with 3.5 mM (control), 0.5 mM (low), or 8 mM (high) K^+^ buffers. Low-chloride buffer was used as a positive control to elevate p-NCC (T58) levels. Graphs show semiquantitative assessment of NCC, PP1, and p-NCC (T58) in NCC immunoprecipitated samples (bottom, right) relative to input. Data are from 3 individual experiments with 3 replicates for each condition (*n* = 9). *P < 0.05, relative to the low-K^+^ condition, by 1-way ANOVA followed by multiple-comparison test. Data are presented as the mean ± SEM. CALNA, calcineurin.

We next sought to test whether PP1A can interact with and regulate NCC directly (Figure 6). We first tested whether recombinant protein phosphatases can dephosphorylate NCC using immunoprecipitated p-NCC as a substrate. As shown in Figure 6A, NCC phosphorylation at T58 was significantly reduced after NCC incubation with PP1A, PP2A, or calcineurin, underscoring the high catalytic activity of serine-threonine protein phosphatases for a broad range of substrates in vitro (45). Because protein phosphatase specificity in vivo is governed by specific binding and substrate/phosphatase colocalization, we tested whether PP1A, PP2A, or calcineurin binds to NCC using glutathione S-transferase (GST) affinity chromatography with purified recombinant PP1A, PP2A, and calcineurin. As shown in Figure 6B, PP1A uniquely interacted with the phospho-regulatory N-terminal domain of NCC (constructed as a GST fusion protein), but not with the negative control GST alone (Figure 6B). Furthermore, neither PP2A nor calcineurin could interact with the NCC N-terminal domain. To determine whether the NCC-PP1A interaction is regulated in renal epithelial cells by potassium, epitope-tagged (FLAG-NCC) was immunoprecipitated with anti-FLAG antibodies from MDCKI-hNCC cells after incubation in control, low-potassium, or high-potassium medium, and immunoprecipitates were probed in immunoblots with NCC, p-NCC, PP1A, or PP2A antibodies and compared with the input. As an additional control, another group of MDCKI-hNCC cells were incubated in low-Cl^–^ medium to corroborate the regulated NCC phosphorylation (26). We found that PP2A did not immunoprecipitate with NCC (data not shown). However, as shown in Figure 6C, incubating cells in a physiological saline solution containing high potassium stimulated PP1-NCC interaction, commensurate with NCC dephosphorylation. Unfortunately, because the canine I1 subunit was not detectable in MDCK cells with the available anti-human/anti-mouse antibodies, we cannot be certain if competitive interactions between I1 and PP1 determine the potassium-dependent binding of PP1 to NCC. Nevertheless, these studies reveal that NCC interaction with PP1A specifically mediated dephosphorylation and that this interaction was enhanced when extracellular potassium was increased.

**Figure 7:**
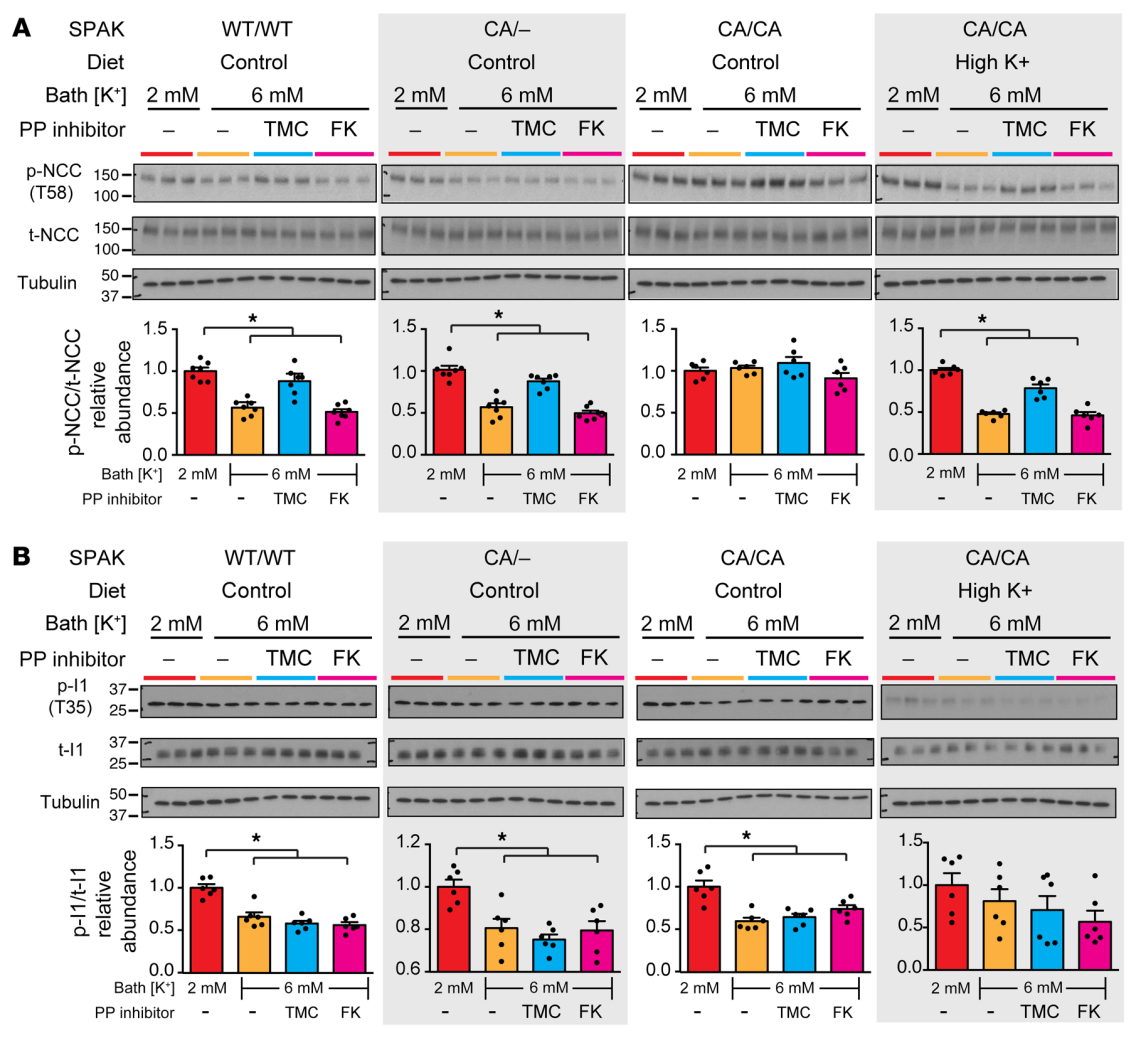
Potassium-dependent activation of PP1 drives rapid dephosphorylation of NCC in the DCT, coincident with dephosphorylation (inactivation) of the PP1-inhibitory subunit I1. (**A**) p-NCC and t-NCC after incubation in control, 2 mM K^+^ bath (red), or 6 mM K^+^ bath containing either vehicle (control, orange), tautomycetin (TMC) (blue), or FK506 (FK) (pink). (**B**) p-I1 and t-I1 as assessed by immunoblotting in kidney slices after incubation in control, 2 mM K^+^ bath (red), or 6 mM K^+^ bath containing either vehicle (control, orange), tautomycetin (TMC) (blue), or FK506 (FK) (pink). Parallel studies were performed on slices from control mice (homozygous for WT SPAK, WT/WT) or heterozygous CA-SPAK mice (CA/–), or homozygous CA-SPAK mice fed the indicated diets. A quantitative summary is shown below the blots. Each lane/dot represents a separate mouse. *n* > 6. *P < 0.05, by 1-way ANOVA with Tukey’s post hoc test. Data are the mean ± SEM.

To test whether PP1 dephosphorylates NCC in response to an acute increase in P_K+_ in the native DCT, potassium-mediated changes in p-NCC were assessed in the presence and absence of protein phosphatase inhibitors in acutely isolated kidney slices (Figure 7A). After preconditioning in a low-potassium medium (2 mM) to maximize NCC phosphorylation, elevation of potassium to 6 mM caused p-NCC to decrease within 60 minutes in control and heterozygous CA-SPAK mice. Furthermore, potassium-dependent NCC dephosphorylation was inhibited by tautomycetin, which is 20–40 times more selective for PP1 than for PP2A (150 nM) (46), but not with the calcineurin inhibitor, FK506 (5 μM) or with 10 nM okadaic acid, which should selectively inhibit PP2A (47) (data not shown). Acute exposure to high potassium did not reduce p-NCC levels in slices isolated from homozygous CA-SPAK mice on the control diet. However, after these mice were adapted to a high-potassium diet for 4 days to allow downregulation of I1, acute exposure to the high-potassium bath caused rapid dephosphorylation of p-NCC, and the response was sensitive to tautomycetin but not FK506.

Parallel measurements of I1 and phosphorylation at the key activation site T35 in I1 (Figure 7B) revealed that elevation of P_K+_ also rapidly stimulated I1 dephosphorylation in WT and CA/CA mice on the control diet. Neither tautomycetin nor FK506 blocked I1 dephosphorylation. In CA/CA mice on the high-potassium diet, I1 basal phosphorylation was low, and it was not possible to detect further potassium-mediated dephosphorylation.

Together, these observations support the conclusion that acute elevation of P_K+_ increased PP1 activity, which drove NCC dephosphorylation. The ability of PP1A to overcome constitutive phosphorylation of NCC in the CA/CA preparations indicates that PP1A directly acted on NCC. Parallel potassium-dependent dephosphorylation of I1 raises the possibility that potassium-induced I1 deactivation may underlie acute PP1 activation.

**Figure 8:**
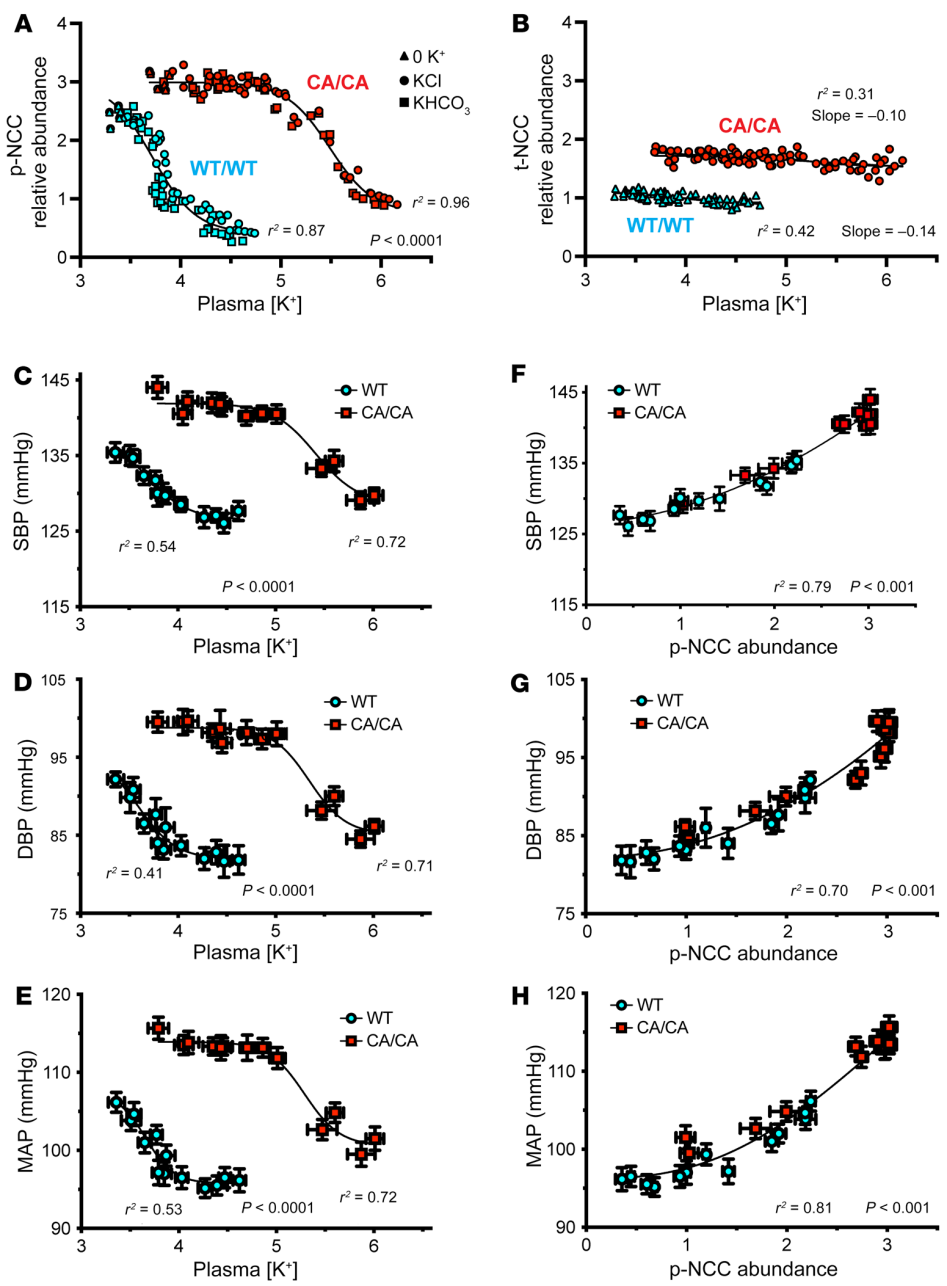
NCC dephosphorylation shapes the BP response to dietary potassium. (**A**) p-NCC (T38) or (**B**) t-NCC relative abundance versus plasma potassium relationships are shown for control mice (WT/WT SPAK, blue) and homozygous CA-SPAK mice (CA/CA, red) fed increasing amounts of potassium chloride (KCl) (circles) or potassium bicarbonate (KHOC_3_) (squares) or a nominally potassium-free diet (triangles). p-NCC and t-NCC values were normalized to control mice on the control diet. Each data point represents a separate mouse. Lines through the p-NCC data are best fits to NCCp(max)/1+ IC_50_/(K^+^)^n^, *n* = –2 IC_50_ of WT/WT, 3.7, CA/CA 5.5 mM K^+^. Lines through the p-NCC data are fit to linear with a small but significant (*P* < 0.0001) non-zero slope. (**C**) Systolic BP (SBP), (**D**) diastolic BP (DBP), and (**E**) mean arterial pressure (MAP) versus plasma potassium relationships for control mice (blue) and CA-SPAK mice (red). Each data point represents the mean ± SEM of BP and plasma potassium levels from more than 5 mice after 4 days on each diet. Lines best fit to data are fit to BP(max)/1+ IC_50_/(K^+^)*^n^*, with *n* fixed at 2 (IC_50_ of WT/WT: SBP, 3.7 mM K^+^; DBP, 3.6 mM K^+^; MAP, 3.7 mM K^+^. IC_50_ of CA/CA: SBP, 5.5 mM K^+^; DBP, 5.3 mM K^+^, MAP 5.3 mM K^+^). *P* values denote the significance of different fits for WT and CA SPAK mice. (**F**) SBP, (**G**) DBP, and (**H**) MAP versus relative p-NCC relationships. Data are derived from data shown in **A**–**E**. Each data point represents the mean ± SEM of BP. Lines best fit to 4 parameter logistic equations. *P* values denote the significance fit as the preferred model over the linear fit.

To explore whether the BP-lowering response to dietary potassium loading is shaped by the potassium-regulated protein phosphatase pathway, we compared NCC phosphorylation and BP in control (WT/WT) and CA/CA mice as dietary potassium content was increased. Mice were subjected to diets with a range of potassium levels (0%, 0.1%, 0.25%, 0.5%, 1.0%, 2.5%, and 5.0%) added as either chloride (KCl) or bicarbonate (KHCO_3_) salts (Figure 8), and BP was measured by radiotelemetry over 4 days after the diet was changed. Within 4 days of increasing the dietary potassium content, plasma potassium levels increased over the physiological range in control mice, whereas CA/CA mice became even more hyperkalemic, causing large decreases in p-NCC (Figure 8A) but not total NCC (t-NCC) (Figure 8B). In control mice, NCC phosphorylation was steeply correlated with P_K+_ between approximately 3 and 4.5 mM (IC_50_ 3.7 mM), with NCC being highly phosphorylated at approximately 3 mM and nearly completely dephosphorylated when potassium exceeded approximately 4.5 mM, regardless of the accompanying anion in the diet. The sigmodal dephosphorylation response was paralleled by similarly-shaped BP responses, which were also independent of the anion (Figure 8, B–D); SBP decreased by 8 mmHg as P_K+_ increased from approximately 3 mM to 4.5 mM (IC_50_ 3.7 mM), but tapered off at higher P_K+_ as NCC became dephosphorylated. By contrast, p-NCC and BP remained elevated in the CA/CA mice at plasma potassium concentrations between approximately 3.5 mM and 5 mM (Figure 8, A and C–E). After P_K+_ exceeded 5 mM, SBP declined steeply by approximately 20 mmHg in the CA/CA mice, correlating with the suppression of PP1 and the inhibitory subunit I1 (see above) and NCC dephosphorylation (IC_50_ 5.5 mM). Strikingly, the rightward shift in the p-NCC–P_K+_ relationship in the homozygous CA-SPAK mice was paralleled by a rightward shift in the BP-P_K+_ relationship (IC_50_ 5.5 mM) (Figure 8, B–D). As a result, the tight correlation between the fall in BP and NCC dephosphorylation in CA/CA mice (Figure 8, F–H) was preserved at the expense of profound hyperkalemia, which drove an increase in PP1 activity to overcome constitutive phosphorylation of NCC.

## Discussion

**Figure 9:**
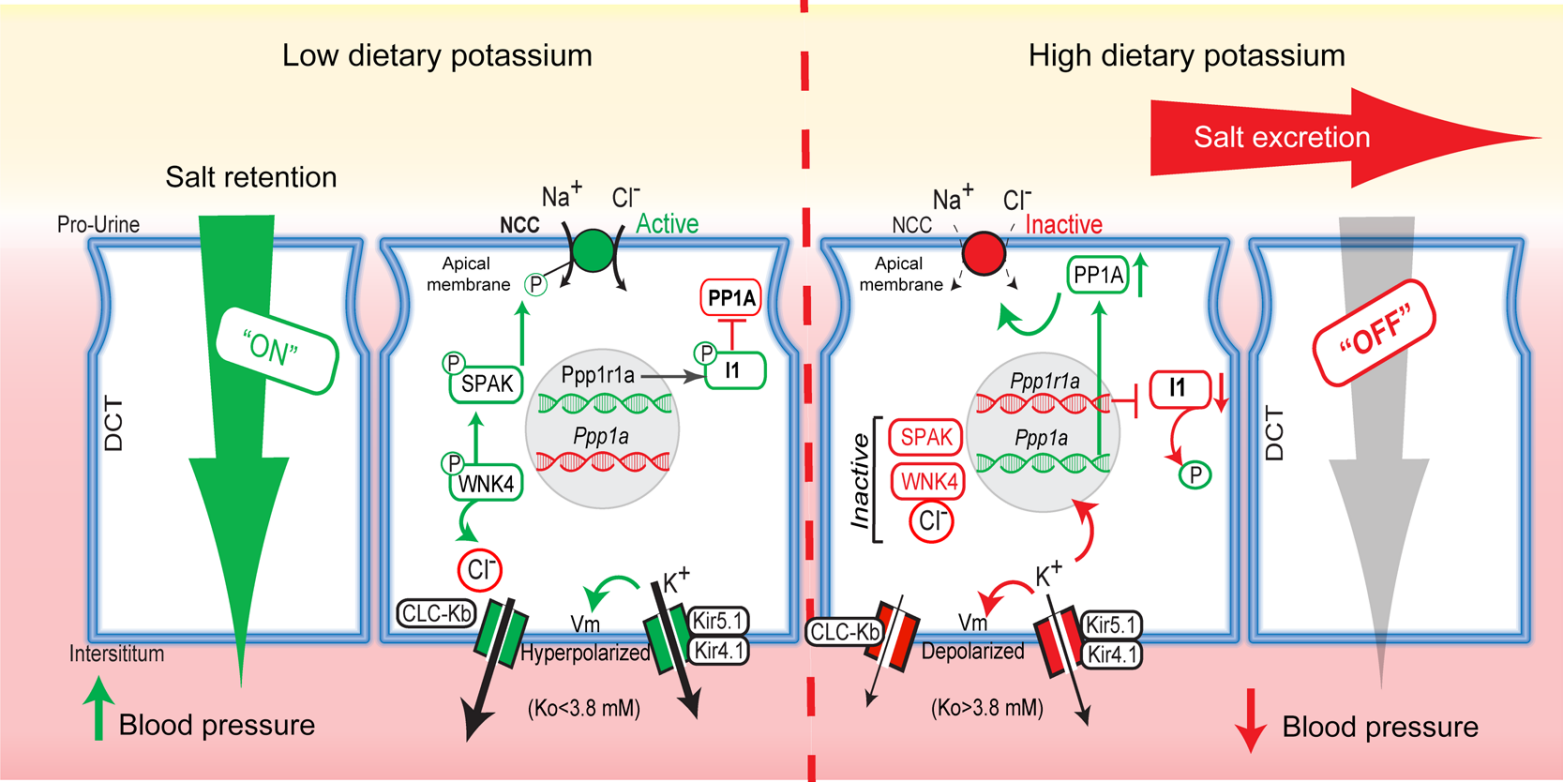
The distal nephron potassium switch controls the activity of the thiazide-sensitive sodium chloride cotransporter NCC over a narrow range of plasma potassium concentrations. Left: As plasma potassium decreases in dietary potassium deficiency, the WNK/SPAK kinase cascade becomes active, and this increases phosphorylation and NCC activity. Under these conditions, the protein phosphatase PP1A is suppressed, and the phosphorylated form of the inhibitory subunit I1 inactivates PP1A. Right: With consumption of a high-potassium diet and physiological increases in plasma potassium, the WNK/SPAK kinase cascade turns off, and the protein phosphatase PP1A becomes active. PP1A binds to NCC directly and inactivates it by dephosphorylation, which leads to diuresis, providing a mechanism to explain how high dietary potassium lowers BP. PP1A binding to NCC is induced rapidly (minutes) after plasma potassium is increased, coinciding with I1 dephosphorylation. With sustained consumption of a high-potassium diet, the *Ppp1a* gene is induced, and the abundance of PP1A protein increases. *Ppp1r1a*, encoding I1, is suppressed under hyperkalemic conditions. Vm, membrane voltage.

Consumption of a potassium-rich diet lowers BP in humans (5, 6) and rodents (26, 48–51). It has been proposed that the response is rooted in the kaliuretic reflexive response of the kidney to a high-potassium diet, which is usually accompanied by increased sodium excretion, a process called potassium-induced natriuresis (52). Growing evidence points to the involvement of a potassium-dependent signaling pathway, the “potassium-switch,” that controls the activity of NCC to maintain sodium-potassium balance. The core elements of the kinase cascade forming the “on” circuit of the switch when dietary potassium is low are well known (29, 53). Indeed, the genetics of FFHt (8) and physiological studies (16, 17, 20, 29, 54, 55) demonstrated that WNK-SPAK kinases activate NCC via phosphorylation. Here, we identify and characterize PP1A and I1 as key components of the mysterious limb of the signaling pathway that rapidly turns NCC off in response to a rise in dietary potassium, causing natriuresis and a decrease in BP (Figure 9). The data reveal that elevation of P_K+_ was sufficient to acutely stimulate PP1A binding and dephosphorylation of NCC. After dietary potassium was increased for several days, induction of the *Ppp1ca* gene increased PP1A, and this enhanced NCC dephosphorylation. Other catalytic PP1 genes — *Ppp1cb*, *Ppp1cc*, and *Ppp1ccb* — are weakly expressed in the DCT (56) and could potentially contribute to NCC regulation alongside Ppp1ca. As plasma potassium levels rose above approximately 5 mM, potassium-dependent suppression of the I1 subunit gene *Ppp1r1* and the cognate protein I1 further activated PP1 to enhance NCC dephosphorylation. These studies add a new dimension to the potassium switch, revealing PP1A and I1 as components of the signaling pathway that turns NCC off directly and reduces BP in response to a potassium-rich diet (Figure 9).

The discovery of the critical “off-circuit” components of the potassium switch reinforces a growing body of data indicating that the PP1A-I1 phosphatase complex acts as a regulatory hub in the DCT, allowing different physiologic stimuli to influence NCC activity. Because the catalytic activities of serine/threonine protein phosphatases are extraordinarily high, increasing the rate of target protein dephosphorylation by 10^21^-fold in vitro (45), specific binding and compartmentalization of catalytic and interacting proteins are required to prevent uncontrolled dephosphorylation and define substrate specificity (44). Recent attention has focused on I1 because it is highly enriched in the DCT (37, 56), and gene-KO studies revealed that it affects NCC phosphorylation and BP (37). Ablation of the *Ppp1r1* gene in mice removes the brake on PP1, causing potent NCC dephosphorylation and hypotension (37). First discovered for its role in glycogen metabolism in the liver (57), it is now well appreciated that I1 and the related members of the PP1 inhibitory subunit family (58) are tightly controlled by phosphorylation in many systems. In the DCT, the inhibitory activity of I1 is increased by PKA-mediated phosphorylation of T35. Activation of Gs protein–coupled receptors stimulates NCC activity through this pathway by increasing cAMP and activating PKA-dependent phosphorylation of I1, which in turn suppresses PP1 to increase NCC phosphorylation (31, 42).

Our studies indicate that potassium activated PP1A-dependent NCC dephosphorylation directly. We found that NCC dephosphorylation occurred within minutes of increasing extracellular potassium in single, isolated DCTs ex vivo, corroborating and extending the findings of recent studies done in heterogenous tubule and kidney splice preparations (32, 35). These observations add to an emerging concept that the DCT acts as a potassium sensor that promptly communicates the effects of dietary potassium to NCC phosphorylation in a cell-autonomous manner through small physiologic changes in extracellular potassium levels. The basolateral membrane Kir4.1/5.1 potassium channel in the DCT (13, 26, 28) transmits the effects of low extracellular potassium to activation of WNK-SPAK (27, 53) kinases and NCC phosphorylation through membrane voltage hyperpolarization (26). Membrane-depolarizing maneuvers, including pharmacologic inhibition of Kir4.1/5.1, stimulate NCC dephosphorylation (26, 28), suggesting that the channel also transmits the effects of high potassium to PP1A activation and NCC dephosphorylation through a membrane voltage-dependent signaling pathway.

Potassium-induced NCC dephosphorylation coincided with I1 dephosphorylation, suggesting that PP1 activation may involve an inhibitory release mechanism. Although calcineurin can dephosphorylate and inactivate I1 in some systems (59), we did not detect any effects of the calcineurin inhibitor FK508 on the potassium-induced dephosphorylation of I1 or NCC. Recent studies in DCT-specific calcineurin regulatory subunit–KO (CnB1-KO) mice indicate that calcineurin only has a relatively minor role in the acute kaliuretic response to an oral potassium bolus (41) and is not required for longer-term, K^+^-dependent phospho-regulation of NCC (41). Because dephosphorylation of I1 was not robust or insufficient to override constitutive phosphorylation of NCC, it seems likely that additional processes drive the activation of PP1A phosphatase activity. In other systems, PP1 is acutely activated through complex combinatorial interactions between PP1, its substrates, and many different regulatory subunits (44).

Extending yeast 2 hybrid–screening studies (31), we found that PP1 directly interacted with the N-terminus of NCC, near the key phosphorylation activation sites, in a potassium-dependent manner. Unlike I1 (60) or the closely related sodium-chloride cotransporters NKCC1 and NKCC2 (61), the NCC N-terminal domain does not harbor a canonical RVxFxD PP1-binding sequence. Noncanonical binding of PP1 I1 to NCC might involve other regulator proteins. There are over 200 known PP1-interacting proteins, and as many as 20 are expressed in the DCT (56). It is possible that one of these swaps for I1 to activate PP1, as has been described in other systems (62). Studies with NKCC1 raise the possibility that PP1 may also associate with SPAK to terminate SPAK phosphorylation of NCC (63), although recent studies hint that an undefined phosphatase inhibits SPAK in the DCT (35).

We found longer-term regulation of PP1Ca and I1 protein abundance and that phosphorylation drove NCC dephosphorylation as mice adapted to the potassium-rich diet. Compared with the acute NCC dephosphorylation in isolated DCTs from mice on a control diet, the curvilinear relationship between potassium and p-NCC shifted leftward in WT mice fed a high-potassium diet, which tracked with the increase in PP1A abundance. The response was especially profound in CA-SPAK mice (CA/CA), in which CA SPAK was overridden and NCC was dephosphorylated after high-potassium diet feeding decreased I1 levels. The changes in PP1A and I1 protein abundance paralleled the changes in transcript abundance, indicative of potassium-dependent induction of *Ppp1Ca* and suppression of *Ppp1r1* genes. Different potassium-dependent signaling pathways probably control *Ppp1ca* and *Ppp1r1* genes as the transcripts are regulated in different directions and ranges of extracellular potassium. Other chronic adaptations of the switch pathway to a high-potassium diet likely contribute to NCC dephosphorylation, including PP1-mediated dephosphorylation and inactivation of WNK4 (64) and SPAK (35). High-potassium diet feeding has also been reported to activate calcineurin activity in the DCT (36), which dampens switch signaling by dephosphorylating residues on the ubiquitin ligase substrate–interacting protein kelch-like family member 3 (KLHL3), leading to increased WNK4 ubiquitination and degradation (36). With chronic consumption of a high-potassium diet, another PP1A-dependent process that Hsp70 facilitates eventually causes the ubiquitination and degradation of NCC (65).

By switching sodium handling in the distal nephron from electroneutral NaCl absorption in the DCT to sodium-potassium exchange in the aldosterone-sensitive distal nephron (ASDN), potassium-induced NCC dephosphorylation induces a thiazide-like natriuresis, which can be accompanied by a decrease in BP (26). However, a causal relationship between potassium-dependent NCC inactivation and BP has been difficult to document. Here, we avoided the stress of prolonged consumption of an extremely high-potassium diet, which can increase BP in mice (54, 66, 67) when potassium is too high or induce diabetes insipidus when it is too low (68), and measured BP over a titration of increased dietary potassium when NCC became acutely dephosphorylated. We found that BP decreased as plasma potassium levels increased, tightly correlating with NCC dephosphorylation over a narrow physiologic range of P_K_ in WT mice. Genetically locking SPAK “on” caused rightward shifts in the BP-P_K_ and p-NCC–P_K+_ relationships, paralleling the suppression of I1 and activation of PP1-mediated NCC dephosphorylation. As a result, we noted a tight relationship between the fall in BP and NCC dephosphorylation over a large dynamic range in WT and homozygous CA-SPAK mice (Figure 7C). The data provide strong evidence for a causal relationship between the acute antihypertensive effects of dietary potassium increases, PP1 induction, and NCC dephosphorylation.

Recent clinical trials indicate that modest substitutions of dietary potassium for sodium can lower BP, benefit cardiovascular health, and reduce mortality in populations consuming high-sodium, low-potassium foods (7, 69). It has been hotly debated whether the benefits are driven primarily by lowering sodium or increasing potassium (70). Our observations indicate that NCC phospho-regulation may be at the nexus of an interaction between the two. We found that dietary potassium induced PP1A expression and NCC dephosphorylation and that it lowered BP even in mice consuming a high-NaCl diet (Supplemental Figure 1). In fact, potassium supplementation blocked the salt-induced elevation in BP and significantly reduced BP more than in mice on the control diet. Thus, PP1A-mediated dephosphorylation of NCC provides a mechanistic explanation for the ability of potassium to reduce salt sensitivity (3).

Our data provide a clue to a puzzling phenotype of FHHt, the development of hyperkalemia before hypertension (71). Adult CA/CA mice recapitulate FHHt in adults (43), as these mice exhibit hyperkalemia, hypertension, and type IV metabolic acidosis (72). We found that the hypertensive phenotype in the adult CA/CA mice diminished once the plasma potassium exceeded approximately 5.5 mM, corresponding to suppression of the inhibitory activity of I1 on PP1 and consequent activation of NCC dephosphorylation. This observation raises the possibility that hyperkalemia may prevent the development of hypertension in FHHt until a second hit disrupts hyperkalemic PP1 activation, allowing the gain-of-function mutations in WNK signaling to fully manifest in maximal NCC phosphorylation. Because the epithelial sodium channel (ENaC) is suppressed as part of a distal tubule remodeling process in homozygous CA-SPAK mice (43), the BP-lowering response to hyperkalemia may also reflect the K^+^-dependent inactivation of NCC on a background of minimal ENaC-mediated Na reabsorption.

The present study has several limitations. First, because the DCT represents a small fraction of the kidney cortex, rare or DCT-specific protein phosphatases may not have been detected by the transcriptomic screen. Future studies with enriched DCT populations or single-cell RNA-Seq will be required to characterize specific DCT transcriptional responses to increased potassium. Furthermore, phosphatases, such as calcineurin, that are chiefly regulated by posttranslational processes would not be captured by the transcriptomic screen. Second, phosphatase inhibitors are not strictly specific. Moreover, regions of the slice preparation may be unavoidably hypoxic and could skew the inhibitor responses. This may explain the variable effects of calcineurin inhibitors on NCC phosphorylation (35, 36, 41). c) Regulation of NCC over a narrow range of P_K_^+^ is likely determined by interactions between WNK-SPAKs and phosphatases, beyond PP1A and I1.

These studies revealed that small increases in P_K+_, which paralleled increased dietary potassium intake, were directly sensed by cells in the DCT and translated to PP1A-mediated dephosphorylation of NCC and a decrease in BP. Shifting signaling of PP1A in favor of activation of WNK-SPAK may provide an improved approach for treating hypertension and hyperkalemia.

## Methods

### Animals.

Kinase-activating mutations were introduced into the SPAK gene, and expression of the CA form of SPAK was specifically targeted to the early DCT using parvalbumin-driven Cre recombinase (Parv-Cre) as previously described (43). For these studies, homozygous CA-SPAK mice (SPAK genotype: CA/CA, Parv-Cre^–/+^) and heterozygous CA-SPAK control mice (SPAK genotype: CA/–, Parv-Cre^–/+^) were compared with control mice (SPAK genotype: WT/WT, Parv-Cre^–/+^). Because CA-SPAK replaces the endogenous SPAK allele, CA-SPAK mice only express CA-SPAK in Parv^+^ cells (e.g., DCT cells) and do not express endogenous SPAK elsewhere (43). Studies were performed using male mice. Heterozygous CA-SPAK mice were generated by breeding homozygous CA-SPAK mice with global SPAK–KO mice (56). All of the mouse strains were on a C57Bl/6J background and backcrossed for more than 10 generations.

### Dietary manipulation.

After mice were 6–7 weeks old, the vivarium house diet was switched to a control diet containing 1% potassium (Envigo, TD.88238). The control diet was matched in composition to the experimental diet except for the potassium content. After acclimation to the control diet for at least 10 days, 8- to 10-week-old mice were randomized to a 4-day dietary protocol for the control diet or to a high-potassium diet (10% KCl, Envigo, TD.10432 or 13.4% KHCO3, Envigo, TD.140044, with both diets containing 5% potassium and the same molar equivalent of anions). At the end of the protocol, animals underwent cervical dislocation with terminal blood and kidney collection.

To investigate how plasma potassium affects NCC activity and BP, mice were fed diets containing different amounts of potassium (0%, 0.1%, 0.25%, 0.5%, 1.0%, 2.5%, 5.0%). Two different potassium salts (KCl and KHCO_3_) were compared and had identical results. Diets were formulated from a base potassium-deficient diet (Envigo, TD.88239) in powered form. The diet was supplemented with varying amounts of KCl and KHCO_3_ to achieve the desired potassium content. In 1 experimental series, matched cohorts of control CA-SPAK mice were fed 1 diet for 4 days and then euthanized to assess P_K+_ levels and harvest kidneys for biochemical experiments. In a parallel series of experiments, BP was measured in matched cohorts of control and CA-SPAK mice using telemetry as described below. BP was measured as mice were moved from the control diet (1% potassium, 4 days) to an experimental potassium diet (4 days), back to the control diet (4 days), to the next experimental potassium diet (4 days). This regiment was repeated until all 7 dietary potassium levels were studied. Mice cycled through the experimental diets from the lowest to the highest potassium levels and always received diets containing the same potassium salt. To test whether the BP response to high potassium was influenced by high-salt (NaCl) consumption, a repeated design was used to measure BP in a cohort of WT mice: (a) in response to a high-potassium diet (5%, 3 days) on the control sodium (0.74%) diet, and then (b) after potassium was returned to control levels for 4 days (washout), and then after (c) dietary sodium intake was increased (2%) for 10 days, and then (d) the BP response to the high-potassium diet was measured again after 3 days.

### Phosphatase screen.

The relative abundance of each protein phosphatase subunit was evaluated by quantitative PCR (qPCR) in control and CA/CA mice on the control or high-potassium diet. mRNA was isolated from the kidney cortex (*n* >6 per genotype per diet) using TRIzol (Thermo Fisher Scientific) and subsequently used for cDNA synthesis (Superscript IV, Thermo Fisher Scientific). A QuantStudio 3 Real-Time qPCR system (Applied Biosystems) along with PowerUp SYBER Green Master Mix and gene-specific primers were used to determine the Ct values. All reactions were performed in triplicate, and the average of these values was normalized to the average Ct value of the housekeeping gene (*Atp5f1*). Melt-curve analysis was performed at the end of the amplification protocol to confirm product specificity. The relative transcript abundance was calculated using the Pfaffl equation (73), a derivation of the ΔΔCt method that accounts for the actual efficiency of doubling within the linear range of amplification. All values are relative to WT mice on the control diet. The potassium-dependent changes across all groups were evaluated by 1-way ANOVA and Tukey’s multiple-comparison test. Genes whose expression levels changed by at least 20% were selected for validation and analysis of correlation with plasma potassium (see Figure 3, B and C).

### Tissue collection.

Animals were anesthetized by intraperitoneal injection with 100 mg/kg ketamine and 10 mg/kg xylazine. Once an animal was unconscious, the kidneys were removed, and the cortex and medulla were separated by free-hand dissection and flash-frozen in liquid nitrogen. Blood samples were collected from the carotid artery. Blood chemistry (Na^+^, K^+^, Cl^–^, HCO_3_^–^, pH, hematocrit, and BUN) were measured using a 100 μL aliquot of whole blood using an i-STAT EC8+ cartridge and an i-STAT1 Handheld Analyzer (Abaxis). The remaining fraction of blood was immediately spun down, and plasma was frozen.

### Ex vivo response to acute changes in extracellular potassium in isolated DCTs ex vivo.

Anesthetized mice were perfused for 5 minutes (8 mL/min) with a modified Ringer’s solution containing 0.1% collagenase type B (MilliporeSigma) and low potassium (2.5 mM K^+^). After perfusion, the left kidney was removed, and the cortex was dissected into 1 mm pieces and incubated at 37°C in the collagenase-Ringer’s solution. After 30 minutes, cortical pieces were transferred to fresh ice-cold modified Ringer’s solution without collagenase, and DCTs were manually dissected from the collagenase-treated tissue at approximately 5°C over 20–30 minutes. After incubation at 37°C for 20 minutes in the 2.5 mM K^+^ Ringer’s solution, isolated DCTs were randomized to Ringer’s solutions containing a range of potassium concentrations (2.0, 3.5, 6.0, 8.0, and 10.0 mM K^+^). After 20 minutes, the tubules were fixed in 2% PFA and processed for immunolabeling using antibodies specific for the T58 phosphorylation site on NCC and for parvalbumin. Appropriate fluorescence-labeled secondary antibodies were subsequently added (Table 1). Images of p-NCC (T58) were captured with a Zeiss LSM 700 confocal microscope, using the same laser power, pinhole, and acquisition settings. K^+^-dependent changes in p-NCC abundance were assessed by comparing pixel intensities along the apical membrane across matched tubules (from the same mouse) in response to K^+^ bath concentrations.

### Ex vivo kidney slices.

Kidneys were removed from anesthetized mice and cut into 2 transverse slices after the capsule was detached. Each half kidney was embedded in 3% agarose at approximately 37°C to 38°C for sectioning. After the agarose solidified, the specimens were mounted onto a Leica BioSystems Vibratome (VT1000S), and 200 μm thick sections were cut. The slices were immediately placed in ice-cold modified Ringer’s solution containing 2 mM potassium (110 mM NaCl, 25 mM NaHCO_3_, 1 mM NaH_2_PO_4_, 2.5 mM CaCl_2_, 1.8 MgCl_2_, and 25 mM glucose). The media were bubbled with 95% CO_2_ and 5% O_2_ for the duration of the experiment and maintained at 35°C. After a 30-minute preincubation period in 2 mM K^+^ to increase NCC phosphorylation, matched slices were randomized and placed in modified Ringer’s solution containing either 2 mM K^+^ or 6 mM K^+^, or 6 mM K^+^ plus tautomycetin (150 nM), or 6 mM K^+^ plus FK506 (tacrolimus, 5 μM). The slices were incubated for an additional 60 minutes and then removed and flash-frozen in liquid nitrogen. To maintain a high level of rigor and reproducibility, different incubations were simultaneously performed in duplicate using specimens obtained from the same mouse, and the different p-NCC levels across the treatment groups were normalized to the control for each mouse.

### Sample preparation for Western blotting.

Frozen mouse kidney cortex or slices were placed in HEENG buffer (20 mM HEPES [pH 7.6], 125 mM NaCl, 1 mM EDTA, 1 mM EGTA, 10% glycerol) containing 1% Triton X-100 and 0.5% SDS with protein and phosphatase inhibitor, sonicated twice on ice using 8-second pulses (20 seconds between pulses with an Ultrasonic Processor XL Sonicator, Heat Systems), incubated at room temperature for 15 minutes, and then slowly rocked at 4°C for 1 hour. The homogenate was centrifuged at 12,000*g* for 10 minutes, and the supernatant was collected. The protein concentration was determined using a bicinchoninic acid protein assay reagent kit (Pierce, Thermo Fisher Scientific). After incubation in Laemmli buffer supplemented with 2-mercaptoethanol, 20 mg kidney protein per sample per well was resolved on precast TGX SDS-PAGE gels (4%–20% gradient, Bio-Rad) and transferred onto membranes using the Bio-Rad TurboBlot system. The membranes were blocked in Tris-buffered saline with 0.1% Tween 20 (TBS-T) containing 5% nonfat dry milk for 1 hour at room temperature, incubated in 5% nonfat dry milk containing a primary antibody (4°C, overnight), washed in TBS-T for 10 minutes (3 times), incubated in 5% nonfat dry milk containing an HRP-conjugated secondary antibody, and then washed for 10 minutes (3 times) in TBS-T. Bound antibodies were then revealed using ECL reagent (Pierce, Thermo Fisher Scientific) and fluorography. Protein quantification was performed by scanning autofluorograms using an Epson Perfection desktop scanner and measuring the integrated density of protein bands using ImageJ software (NIH). Bands were measured in the linear range of the fluorographic signal. Duplicate gels were processed and developed in parallel for the detection of the loading control (tubulin). Unless otherwise stated, each protein signal was divided by its tubulin signal to yield a tubulin-normalized signal.

### Immunolocalization and image analysis.

Anesthetized mice were fixed by perfusion with 2% paraformaldehyde in PBS via the left ventricle for 5 minutes at room temperature. The kidneys were then removed and fixed for an additional 24 hours at 4°C, rinsed in PBS, and embedded in paraffin. Cross-sections (3 μm thick), cut at the papilla, were picked up on chrome-alum gelatin–coated glass coverslips and dried on a warming plate. The sections were then deparaffinized in 2 xylene baths and 2 absolute ethanol baths, for 5 minutes each, and rehydrated in a graded ethanol series to distilled water. For epitope retrieval, the coverslips were placed in a pH 8 aqueous solution containing Tris (1 mM), EDTA (0.5 mM), and SDS (0.02%). The retrieval solution was heated to boiling in a microwave oven, transferred to a conventional boiling water bath for 15 minutes, and then allowed to cool to room temperature before the sections were thoroughly washed in distilled water to remove the SDS. Sections were preincubated for 30 minutes with Image-iT blocking solution (Invitrogen, Thermo Fisher Scientific), rinsed in PBS, and then preincubated for an additional 30 minutes in a solution of 2% BSA, 0.2% fish gelatin, 5% normal donkey serum, and 0.2% sodium azide in PBS. Tissues were rinsed thoroughly with TBS to remove PBS and were then incubated with specific antibodies for 12–18 hours in a humid chamber at 4°C in TBS containing 1% BSA, 0.2% fish gelatin, 0.1% Tween 20, 10 mM CaCl_2_, and 0.2% sodium azide. After thorough washing in a high-salt wash (incubation medium plus added NaCl at 0.5 M), Alexa Fluor 405–, 488–, 568–, and 649–conjugated donkey anti-mouse, anti-rabbit, anti-chicken, and anti–guinea pig secondary IgG antibodies (The Jackson Laboratory) were used to visualize specific target proteins. Quantitative analysis of confocal images (fluorescence intensity and colocalization) was performed using ImageJ by a trained investigator who was blinded as to identity of the sample groups. The average pixel intensity was evaluated in DCT cells, excluding the nuclei, and the local background was subtracted. At least 30 cells from 8 tubules per mouse were counted.

### GST-NCC fusion protein interaction with Ppp1ca.

The cytoplasmic amino terminus of rat NCC (NP_062218) encoding amino acids 1–133 was cloned into a pGEX4T1 vector to produce a GST-(N) NCC N-terminal fusion construct. This, or the empty pGEX4T1 vector, was transformed into C43 (DE3) *E*. *coli* bacteria, which were grown in 200 mL LB^+^ ampicillin (37°C) in a shaking incubator, and GST proteins were recovered by glutathione chromatography from cell homogenates solubilized in PBS buffer and 1% Triton X-100 and centrifuged for 15 minutes at 15,000*g*. The beads were washed 3 times with PBS to generate a 50% purified protein slurry. For GST-pulldown studies, 5 mg GST-(N) NCC or GST protein alone was combined with 2–10 μg recombinant α catalytic subunit of rabbit protein phosphatase 1 (Ppp1ca, MilliporeSigma, P7937), PP2A (Origene, catalog TP760699), or calcineurin (PPP3CA Origene, catalog TP762295) reconstituted in a solution of 250 mM NaCl, 50 mM imidazole, pH 7.4, 2 mM DTT, 1 mM EDTA, 2 mM MnCl_2_, 0.025 % Tween 20, 100 mg/mL trehalose, and 20% (v/v) glycerol. After a 12- to 18-hour incubation in PBS with 0.5 mM TCEP buffer at 4°C, beads were collected, washed 3 times with 1× PBS plus 0.5 mM TCEP buffer to remove any unbound protein, and bound protein was eluted with 5× SDS loading buffer (30 min at room temperature) and evaluated by Western blotting.

### In vitro phosphatase experiment.

MDCKI-hNCC cells (74) were grown in DMEM High Glucose with 10% DBS in T75 culture flasks. Cells were induced with 10 μg/mL tetracycline HCl for approximately 18 hours prior to the experiment. Cells were washed twice in isotonic buffer (135 mM NaCl, 5 mM KCl, 1 mM CaCl_2_, 1 mM MgCl_2_, 1 mM Na_2_HPO_4_, 1 mM Na_2_SO_4_, 15 mM sodium HEPES, pH 7.4) before maximizing NCC phosphorylation by stimulation with hypotonic low-chloride buffer (67.5 mM sodium gluconate, 2.5 mM potassium gluconate, 0.5 mM CaCl_2_, 0.5 mM MgCl_2_, 1 mM Na_2_HPO_4_, 1 mM Na_2_SO_4_, 7.5 mM sodium HEPES, pH 7.4) for 20 minutes at 37°C. Cells were subsequently washed in ice-cold PBS (pH 7.5), scraped and sonicated in IP lysis buffer (135 mM NaCl, 20 mM Tris [pH 7.4], 1% NP-40, 5 mM EDTA) with 5 μg/mL leupeptin, 100 μg/mL Pefabloc, and PhosSTOP phosphatase inhibitor tablets (Roche Diagnostics). After centrifugation at 10,000*g* for 10 minutes at 4°C, the supernatant was subjected to IP using 1/8 volume of Anti-FLAG M2 Affinity Gel Beads (MilliporeSigma) at 4°C overnight with rotation. Resin was washed 3 times with lysis buffer (with inhibitors) and eluted with 200 μg/mL FLAG peptide solution (GenScript) in TBS (10 mM Tris-HCl, 150 mM NaCl, pH 7.4). Excess FLAG peptides were removed from the IP eluate using Vivacon 500 spin columns (30,000 molecular weight cutoff [MWCO], Viva Products). Eluate was incubated with either (a) 10 units of Protein Phosphatase 1 Catalytic Subunit, α-isoform (MilliporeSigma, catalog P7937; 6123.08 units/mg) and 1 mM MnCl_2_ in 1× PP1 reaction buffer (10 mM NaCl, 5 mM imidazole [pH 7.4], 0.2 mM DTT, 2.5‰Tween 20); (b) 25 ng PP2Aα (SignalChem, catalog P16-20BH) in 1× PP2 reaction buffer (25 mM HEPES [pH 7.2], 50 mM NaCl, 2.5 mM EDTA, 50 mM imidazole, 0.2% 2-mercaptolethanol, 65 ng/μL BSA); or (c) 10 units of PP3 (MilliporeSigma, catalog C1907), 1 mM MnCl_2_ and 10 μg/mL calmodulin (MilliporeSigma, catalog P0270) in 1× PP3 reaction buffer (50 mM Tris-HCl [pH 7.0], 50 μM CaCl_2_, 50 μg/mL BSA) at 30°C for 30 minutes. Concentrated Laemmli sample buffer with 15 mg/mL DTT (final concentration) was added to each reaction and heated for 15 minutes at 60°C. Subsequently, 15 μL of the reactions was used for immunoblotting with mouse monoclonal anti-NCC antibody (65) or an anti–p-NCC (T58) antibody (75) using standard procedures as described above.

### NCC IP.

MDCKI-hNCC cells (74) were grown in DMEM high glucose with 10% FBS on semipermeable supports (0.4 μm pore, Corning) coated with basement membrane extract (Cultrex Basement Membrane Extract, PathClear, R&D Systems) until confluent. NCC expression was induced by treating cells with 10 μg/mL tetracycline HCl for approximately 18 hours prior to the experiment. Cells were washed twice in DMEM containing 3.5 mM KCl (110 mM NaCl, 26 mM NaHCO_3_, 1.8 mM CaCl_2_, 0.81 mM MgCl_2_, 2.48 μM Fe(NO_3_)3, 0.91 mM Na_2_HPO_4_, 25 mM glucose, 1 mM sodium pyruvate, 0.4 mM glycine, 4 mM l-glutamine, MEM vitamin solution (Thermo Fisher Scientific), and MEM amino acids (Thermo Fisher scientific). Subsequently, cells were incubated in DMEM containing 0.5 mM KCl (low), 3.5 mM KCl (control), or 8 mM KCl (high) for 15 minutes at 37°C. In some experiments, hypotonic low-chloride buffer (67.5 mM sodium gluconate, 2.5 mM potassium gluconate, 0.5 mM CaCl_2_, 0.5 mM MgCl_2_, 1 mM Na_2_HPO_4_, 1 mM Na_2_SO_4_, 7.5 mM sodium HEPES, pH 7.4) was used as a control to elevate levels of p-NCC (T58). Media were removed, and cells were lysed in 500 μL of 135 mM NaCl, 20 mM Tris (pH 7.4), 1% NP-40, and 5 mM EDTA containing cOmplete Mini Protease Inhibitor and PhosSTOP phosphatase inhibitor tablets (Roche Diagnostics). Lysates were briefly sonicated and centrifuged at 10,000*g* for 5 minutes at 4°C, before 50 μL supernatant was removed as a total lysate sample. The remaining supernatant was subjected to IP as described previously with minor changes (31, 65). Supernatants were incubated with 40 μL Anti-FLAG M2 Affinity Gel beads (MilliporeSigma) at 4°C overnight with rotation. Resin was washed 3 times with lysis buffer containing protease and phosphatase inhibitors, and immunoprecipitated proteins were eluted with 200 μg/mL FLAG peptide solution (GenScript) in 10 mM Tris-HCl, 150 mM NaCl, pH 7.4. Gel samples were prepared by addition of Laemmli gel sample buffer containing 15 mg/mL DTT (final) and heated for 15 minutes at 60°C. Samples were immunoblotted using standard techniques and probed using a mouse monoclonal anti-NCC antibody (65), an anti–p-NCC (T58) antibody (75), a mouse monoclonal anti-PP1α antibody (Thermo Fisher Scientific, catalog 43-8100), and polyclonal anti–proteasome 20S antibodies (Santa Cruz Biotechnology, catalog sc-67339). In some experiments, nontetracycline-induced samples (containing no NCC) were used as control for the interaction between NCC and PP1, with minor unspecific binding of PP1 to the agarose beads occasionally observed, but this remained constant with different K^+^ concentrations.

### BP measurements.

BP was measured in conscious control mice and CA-SPAK mice using a Data Sciences International (DSI) telemetry-based system. The catheters from PA-C10 telemeters were surgically inserted into the internal carotid artery as previously described (43, 56). Mice were allowed to recover from surgery for 7 days before measurements were done. Dataquest ART 4.2 software was used to create a sampling program that measured the BP of each animal for 1 minute every 5 minutes. For these studies, the average SBP, DBP, and MAP were assessed over the 12-hour active period when lights were off (6 pm to 6 am), on the fourth day of each dietary intervention.

### Statistics.

Data are presented as the mean ± SEM. Statistical analysis was performed using GraphPad Prism 9 (GraphPad Software). When a single variable was compared between 2 groups, the data were analyzed using an unpaired, 2-tailed Student’s *t* test. ANOVAs were used when comparing 3 or more groups. The influence of 1 independent variable (e.g., diet or genotype) or 2 different independent variables (e.g., diet and genotype) on 1 continuous dependent variable (e.g., metabolic data or gene/protein abundance) was assessed using either 1-way (single-variable) or 2-way (2-variable) ANOVAs. Correction for multiple comparisons was obtained using Tukey’s test. Values were considered significant when the *P* value was less than or equal to 0.05.

### Data availability.

Data will be made available upon request. Values for all data points in graphs can be found in the Supplemental Supporting Data Values file.

### Study approval.

All animal experiments were approved by the IACUCs of Johns Hopkins University and the University of Maryland School of Medicine.

## Author contributions

PRG designed research studies, conducted experiments, acquired data, analyzed data, and wrote the manuscript. AT conducted experiments and acquired and analyzed data. LLR designed research studies, conducted experiments, and acquired data and analyzed data. BYK conducted experiments and acquired data and analyzed data. DL conducted experiments and acquired data. EJD provided reagents and advice and edited the manuscript. RAF designed research studies, provided reagents, and wrote the manuscript. PAW oversaw the project, managed the design of research studies, analyzed data, and wrote the manuscript.

## Supplementary Material

Supplemental data

Supporting data values

## Figures and Tables

**Table 1:**
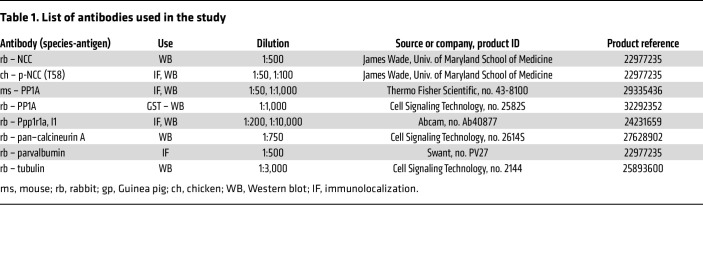
List of antibodies used in the study

## Version 1 09/07/2023

In-Press Preview

## Version 2 11/01/2023

Electronic publication
