# Supplementary material for: Dietary potassium stimulates Ppp1Ca-Ppp1r1a dephosphorylation of kidney NaCl cotransporter and reduces blood pressure
Source: J Clin Invest. 2023 Nov 1;133(21):e158498. doi: 10.1172/JCI158498 (PMC10617769; doi:10.1172/JCI158498)
Supplement: Supplemental data [file jci-133-158498-s083.pdf]

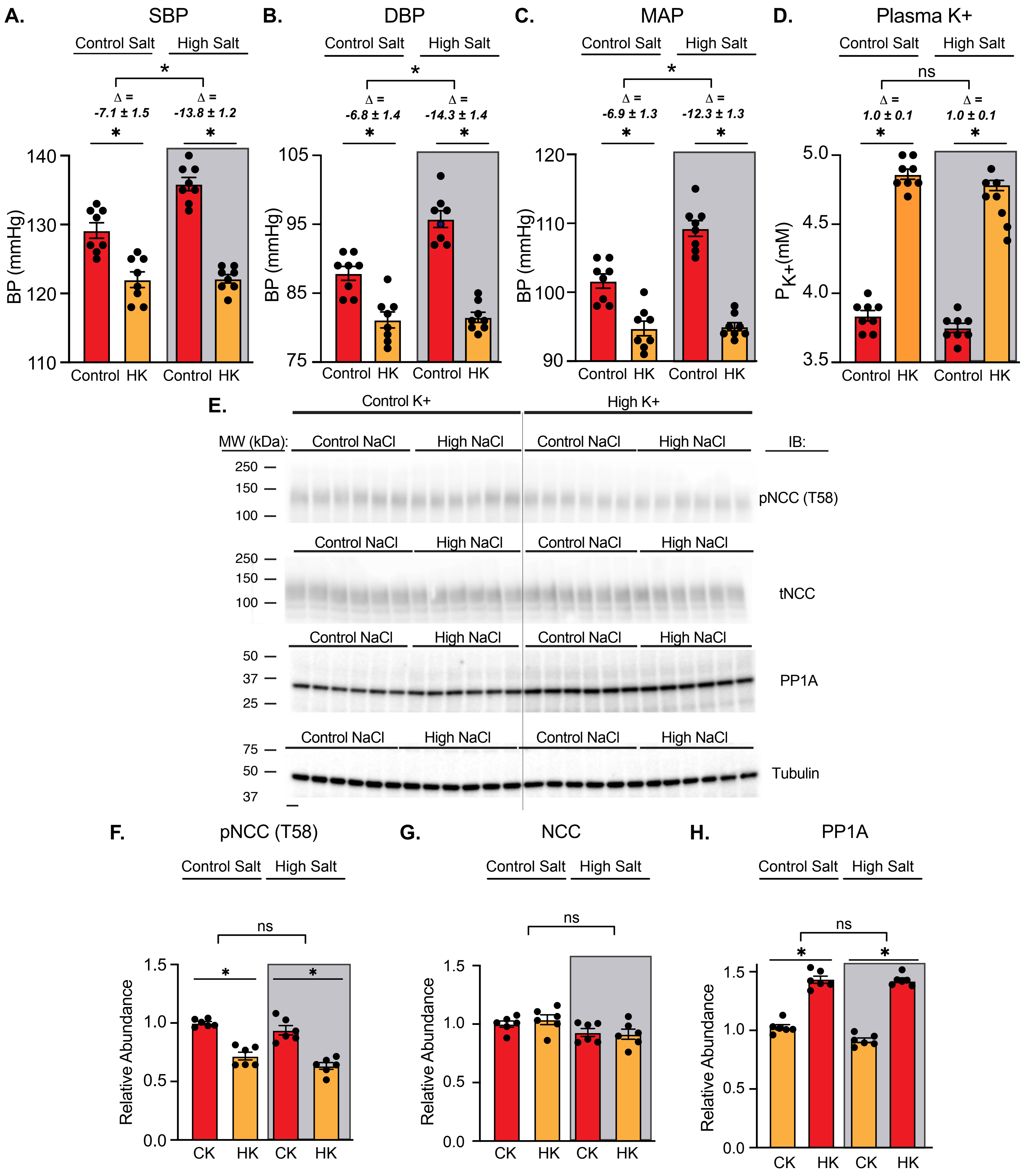

Supplemental Figure 1. High dietary potassium (HK) induces PP1A and reduces NCC phosphorylation and BP on high dietary salt background.
A. Systolic Blood Pressure (SBP), B. Diastolic Blood Pressure (DBP), C. Mean Arterial Blood Pressure (MAP) were measured in a cohort of mice (N=6): 1) on control diet and control potassium (CK), and then in response to high potassium diet (HK). After high potassium washout (4 days), BP was measured on high salt diet (2%, 10d, High Salt, CK), and the high potassium response was measured after 3 days on the high sodium diet. BP and potassium induced changes in BP are compared, * P <0.05, Repeated Measure One Way AVOVA, Tukey's.
D. plasma potassium (K+), F. Phospho-NCC (T58), G. total NCC, and H. PP1A protein abudnace.E. Immunoblot of F, G, H. * at least p<0.05, N= 6

Supplemental Table 1

Genotype	WT/WT Mice				CA/CA Mice			
	Control Diet (n=7)		High K+ Diet (n=7)		Control Diet (n=7)		High K+ Diet (n=7)	
Gene	Norm Ave	SEM	Norm Ave	SEM	Norm Ave	SEM	Norm Ave	SEM
Ppm1a	1.000	0.015	0.988	0.021	1.009	0.017	1.028	0.025
Ppm1b	1.000	0.016	0.991	0.014	0.969	0.019	0.992	0.016
Ppm1d	1.000	0.015	1.046	0.020	1.004	0.011	1.013	0.015
Ppm1e	1.000	0.013	1.016	0.025	1.000	0.011	0.996	0.009
Ppm1f	1.000	0.011	1.008	0.025	1.016	0.016	1.007	0.010
Ppm1g	1.000	0.016	0.962	0.018	0.970	0.019	0.972	0.012
Ppm1h	1.000	0.025	0.983	0.011	0.986	0.020	0.981	0.010
Ppm1j	1.000	0.023	0.953	0.027	0.991	0.017	0.977	0.009
Ppm1k	1.000	0.015	0.990	0.016	1.004	0.019	1.004	0.015
Ppm1l	1.000	0.013	0.995	0.011	0.986	0.017	0.990	0.012
Ppm1m	1.000	0.013	0.999	0.023	0.985	0.008	0.991	0.014
Ppm1n	1.000	0.029	1.031	0.016	0.997	0.016	1.001	0.012
Ppme1	1.000	0.024	1.003	0.018	0.994	0.021	0.996	0.012
Ppp1ca	1.000	0.005	1.459*	0.050	1.773*	0.017	1.804*	0.014
Ppp1cb	1.000	0.011	1.010	0.016	0.989	0.010	1.026	0.016
Ppp1cc	1.000	0.013	1.009	0.011	1.002	0.021	0.998	0.015
Ppp1r1a	1.000	0.010	1.001	0.007	0.863*	0.015	0.724*	0.018
Ppp1r1b	1.000	0.016	1.019	0.018	1.007	0.017	1.013	0.012
Ppp1r2	1.000	0.013	0.989	0.026	1.010	0.014	1.019	0.013
Ppp1r3a	1.000	0.029	1.004	0.015	0.982	0.011	0.998	0.015
Ppp1r3b	1.000	0.025	1.024	0.024	1.008	0.021	1.001	0.012
Ppp1r3c	1.000	0.027	0.947	0.012	1.008	0.015	1.020	0.016
Ppp1r3d	1.000	0.023	0.957	0.025	0.962	0.007	0.953	0.011
Ppp1r3e	1.000	0.016	1.028	0.023	1.028	0.019	1.021	0.020
Ppp1r7	1.000	0.013	0.950	0.027	0.914*	0.010	0.854*	0.022
Ppp1r8	1.000	0.028	0.995	0.018	0.979	0.022	0.995	0.017
Ppp1r9a	1.000	0.021	1.027	0.019	1.025	0.025	1.005	0.018
Ppp1r9b	1.000	0.025	1.016	0.014	0.978	0.019	0.998	0.017
Ppp1r10	1.000	0.013	0.986	0.012	1.060	0.025	1.103*	0.008
Ppp1r11	1.000	0.022	0.973	0.023	0.989	0.010	0.996	0.012
Ppp1r12a	1.000	0.018	1.020	0.018	0.998	0.023	0.991	0.013
Ppp1r12b	1.000	0.028	0.965	0.017	1.049	0.022	1.028	0.010
Ppp1r12c	1.000	0.015	1.001	0.029	1.030	0.018	1.010	0.011
Ppp1r13b	1.000	0.010	0.999	0.027	0.994	0.011	1.018	0.020
Ppp1r14a	1.000	0.021	1.019	0.019	1.008	0.022	1.006	0.015
Ppp1r14b	1.000	0.026	0.986	0.023	0.974	0.015	0.998	0.013
Ppp1r14c	1.000	0.024	1.054	0.024	1.027	0.011	1.016	0.013
Ppp1r14d	1.000	0.023	1.009	0.026	0.971	0.024	0.995	0.012
Ppp1r15a	1.000	0.016	0.988	0.026	0.994	0.012	1.017	0.017
Ppp1r15b	1.000	0.021	1.004	0.024	0.993	0.027	1.007	0.010
Ppp1r16a	1.000	0.020	0.924*	0.020	0.958	0.010	0.963	0.010
Ppp1r16b	1.000	0.018	0.981	0.025	0.992	0.010	0.993	0.010
Ppp1r17	1.000	0.011	0.974	0.011	0.974	0.016	0.997	0.013
Ppp1r18	1.000	0.017	0.991	0.028	0.999	0.010	0.993	0.017
Ppp1r21	1.000	0.020	1.022	0.010	1.000	0.026	1.010	0.012
Ppp1r27	1.000	0.008	1.014	0.026	0.997	0.027	1.004	0.012
Ppp1r32	1.000	0.015	0.984	0.011	0.979	0.015	1.037	0.020
Ppp1r35	1.000	0.024	0.973	0.021	1.015	0.027	1.012	0.018
Ppp1r36	1.000	0.013	0.993	0.022	1.007	0.017	1.005	0.013
Ppp1r37	1.000	0.023	0.988	0.010	0.965	0.027	0.986	0.009
Ppp1r42	1.000	0.027	0.953	0.025	0.986	0.010	1.002	0.015
Ppp2ca	1.000	0.030	0.987	0.037	1.032	0.040	1.035	0.033
Ppp2cb	1.000	0.024	0.953	0.010	0.963	0.007	1.013	0.012
Ppp2r1a	1.000	0.011	1.043	0.021	1.075*	0.014	1.065*	0.009
Ppp2r1b	1.000	0.024	1.054	0.021	1.017	0.025	1.015	0.009
Ppp2r2a	1.000	0.011	1.007	0.012	1.000	0.010	1.005	0.011
Ppp2r2b	1.000	0.026	1.000	0.014	0.994	0.015	1.006	0.014
Ppp2r2c	1.000	0.028	1.022	0.013	1.005	0.015	0.998	0.014
Ppp2r2d	1.000	0.023	1.012	0.028	1.025	0.011	1.022	0.010
Ppp2r3a	1.000	0.024	0.973	0.025	0.971	0.018	0.963	0.009
Ppp2r3c	1.000	0.022	1.001	0.020	0.990	0.011	1.002	0.012
Ppp2r4	1.000	0.020	1.031	0.019	1.028	0.014	1.026	0.013
Ppp2r5a	1.000	0.017	0.984	0.017	0.986	0.012	0.994	0.008
Ppp2r5c	1.000	0.013	1.025	0.023	1.006	0.009	1.009	0.009
Ppp2r5e	1.000	0.022	0.942	0.028	0.967	0.012	0.979	0.015
Ppp3ca	1.000	0.017	1.113*	0.022	1.178*	0.005	1.203*	0.015
Ppp3cb	1.000	0.015	0.998	0.027	0.989	0.011	0.995	0.011
Ppp3cc	1.000	0.019	0.975	0.025	0.979	0.015	0.986	0.010
Ppp3r1	1.000	0.019	0.967	0.012	0.969	0.012	0.962	0.007
Ppp3r2	1.000	0.023	0.980	0.009	0.989	0.017	0.990	0.011
Ppp4c	1.000	0.025	1.000	0.018	1.027	0.013	1.006	0.014
Ppp4r1	1.000	0.011	0.984	0.025	1.000	0.014	1.001	0.014
Ppp4r2	1.000	0.014	1.015	0.026	0.978	0.009	0.990	0.010
Ppp4r4	1.000	0.014	0.983	0.026	0.988	0.010	0.986	0.012
Ppp5c	1.000	0.016	0.999	0.029	0.995	0.019	1.001	0.010
Ppp6c	1.000	0.025	1.035	0.020	1.026	0.016	1.008	0.015
Ppp6r1	1.000	0.020	0.979	0.023	0.968	0.017	0.994	0.014
Ppp6r2	1.000	0.012	1.016	0.022	0.999	0.010	0.988	0.015
Ppp6r3	1.000	0.024	1.001	0.017	0.988	0.017	0.992	0.018

Supplemental Acknowledgments

LeDucq Potassium in Hypertension Research Network of Excellence

North American Coordinator:

Paul A. **Welling**
Johns Hopkins School of Medicine
Department of Medicine (Nephrology)
Baltimore, MD

European Coordinator:

Robert A. **Fenton**
Aarhus University,
Department of Biomedicine
Aarhus, Denmark

Network Members:

David H. **Ellison**<br>Oregon Clinical and Translational Research Institute<br>Oregon Health and Science University<br>Portland, OR, USA	Eric **Delpire**<br>Vanderbilt University Medical Center<br>Department of Anesthesiology<br>Nashville, TN USA
Olivier **Staub**<br>University of Lausanne<br>Department of Pharmacology & Toxicology<br>Lausanne, Switzerland	Michael **Stowasser**<br>University of Queensland School of Medicine<br>Hypertension Unit<br>Brisbane, Australia
